# A cohesin‐associated gene score may predict immune checkpoint blockade in hepatocellular carcinoma

**DOI:** 10.1002/2211-5463.13474

**Published:** 2022-09-02

**Authors:** Cui‐Zhen Liu, Jian‐Di Li, Gang Chen, Rong‐Quan He, Rui Lin, Zhi‐Guang Huang, Jian‐Jun Li, Xiu‐Fang Du, Xiao‐Ping Lv

**Affiliations:** ^1^ Department of Medical Oncology The First Affiliated Hospital of Guangxi Medical University Nanning China; ^2^ Department of Pathology The First Affiliated Hospital of Guangxi Medical University Nanning China; ^3^ Department of General Surgery The Second Affiliated Hospital of Guangxi Medical University Nanning China; ^4^ Department of Gastroenterology The First Affiliated Hospital of Guangxi Medical University Nanning China

**Keywords:** cohesin‐associated gene score, immunotherapy, MEGENA, STAG1, TME

## Abstract

Stromal antigen 1 (STAG1), a component of cohesion, is overexpressed in various cancers, but it is unclear whether it has a role in the transcriptional regulation of hepatocellular carcinoma (HCC). To test this hypothesis, here, we screened global HCC datasets and performed multiscale embedded gene co‐expression network analysis to identify the potential functional modules of differentially expressed STAG1 co‐expressed genes. The putative transcriptional targets of STAG1 were identified using chromatin immunoprecipitation followed by high‐throughput DNA sequencing. The cohesin‐associated gene score (CAGS) was quantified using the The Cancer Genome Atlas HCC cohort and single‐sample gene set enrichment analysis. Distinct cohesin‐associated gene patterns were identified by calculating the euclidean distance of each patient. We assessed the potential ability of the CAGS in predicting immune checkpoint blockade (ICB) treatment response using IMvigor210 and GSE78220 cohorts. STAG1 was upregulated in 3313 HCC tissue samples compared with 2692 normal liver tissue samples (standard mean difference = 0.54). A total of three cohesin‐associated gene patterns were identified, where cluster 2 had a high *TP53* mutated rate and a poor survival outcome. Low CAGS predicted a significant survival advantage but presaged poor immunotherapy response. Differentially expressed STAG1 co‐expression genes were enriched in the mitotic cell cycle, lymphocyte activation, and blood vessel development. *PDS5A* and *PDGFRA* were predicted as the downstream transcriptional targets of STAG1. In summary, STAG1 is significantly upregulated in global HCC tissue samples and may participate in blood vessel development and the mitotic cell cycle. A cohesin‐associated gene scoring system may have potential to predict the ICB response.

## Graphical abstract

According to the relative expression levels of 17 cohesin‐associated gene (i.e., *RAD21*, *SMC1A*, *SMC3*, *STAG1*, *STAG2*, *CDCA5*, *MAU2*, *NIPBL*, *PDS5A*, *PDS5B*, *WAPL*, *AURKB*, *CDK1*, *ESPL1*, *PLK1*, *PTPA*, and *SGO1*), a cohesin‐associated gene score may be calculated to portray the molecular characteristics of hepatocellular carcinoma. The cohesin‐associated gene score may be helpful for predicting immune checkpoint blockade in hepatocellular carcinoma.

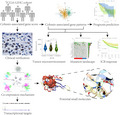

**Abbreviations**
CAG: cohesin‐associated gene
CAGS: cohesin‐associated genes scores
ChIP‐seq: chromatin immunoprecipitation sequencing
CIBERSORT: cell type identification by estimating relative subsets of RNA transcripts
CR: complete remission
ESTIMATE: estimation of STromal and immune cells in MAlignant tumor tissue using expression data
HCC: hepatocellular carcinoma
ICB: immune checkpoint blockade
MEGENA: multiscale embedded gene co‐expression network analysis
PD: progressive disease
PR: partial remission
SD: stable disease
SMD: standard mean difference
ssGSEA: single‐sample gene set enrichment analysis
STAG1: stromal antigen 1
WGCNA: weighted gene co‐expression network analysis

Hepatocellular carcinoma (HCC), one of the most pernicious malignancies in the digestive system, comprises approximately 80% of liver cancer cases and poses a significant threat to the global population [1]. Risk factors for HCC vary from region to region, and nonalcoholic fatty liver disease has become the fastest‐growing cause of HCC in the United States (US) and the United Kingdom [2]. Although surgical resection, radiofrequency ablation, and transcatheter arterial chemoembolization have been used to treat HCC patients, there is a dearth of effective therapeutic strategies for progressed HCC [3, 4, 5]. Immunotherapy exhibits impressive prospects in treating HCC patients [6]; however, significant heterogeneity exists when responding to immune checkpoint blockade (ICB) treatment [7, 8]. Therefore, there is an urgent need to identify more effective indicators for predicting the response to ICB treatment [9]. Moreover, the ultimate pathogenesis of HCC must be identified, and more effort must be made to treat recurrent and metastatic HCC patients [10, 11].

The cohesin core subunit, stromal antigen 1 (STAG1), is encoded by the *STAG1* gene, which belongs to the sister chromatid cohesion protein 3 family, and is ubiquitously expressed in the nucleus [12]. It is known that the cohesin protein complex is indispensable for sister chromatid cohesion; it also plays important roles in transcriptional regulation in addition to chromosome maintenance [13]. Interestingly, STAG1 exerts its function by binding to cohesin with the aid of the RAD21 cohesin complex component, thus generating a platform for the binding of other regulatory cohesin subunits [14]. It has been reported that cohesin can promote DNA damage repair [15], and cohesin mutations are commonly detected in cancers [16, 17, 18]. Moreover, inactivated STAG1 was found to attenuate the proliferation of bladder cancer and sarcoma cells [19]. Nonetheless, the role of the STAG1 transcriptional factor (TF) in HCC remains unknown.

Given the lack of research on STAG1, this study focused on identifying novel cohesin‐associated HCC phenotypes and exploring the underlying transcriptional regulatory mechanism, thus providing avenues for treating HCC patients.

## Materials and methods

### Cohesin‐associated gene signatures in the cancer genome atlas liver HCC cohort

The design route of this research study is displayed in Fig. S1. Level three fragments per kilobase of transcript per million fragments mapped (FPKM) data were downloaded from The Cancer Genome Atlas (TCGA) and were transformed into transcripts per million (TPM) values. As a pivotal component of cohesin, STAG1 exerts its function with other cohesin‐associated genes (CAGs). Therefore, the expression data of 17 CAGs were abstracted, including five subunits (*RAD21*, *SMC1A*, *SMC3*, *STAG1*, and *STAG2*), six regulatory factors (*CDCA5*, *MAU2*, *NIPBL*, *PDS5A*, *PDS5B*, and *WAPL*), and six regulation controlling factors (*AURKB*, *CDK1*, *ESPL1*, *PLK1*, *PTPA*, and *SGO1*) [20]. Single‐sample gene set enrichment analysis (ssGSEA) was used to quantify the cohesin‐associated signatures in each of the HCC patients, which were defined as the CAG scores (CAGS).

### Identification of the CAG patterns in HCC patients

Principal component analysis (PCA) was conducted to determine the ability of the CAGs to discriminate between HCC and the adjacent normal liver tissue samples. HCC tissue samples were assigned to low, moderate, and high CAGS clusters by calculating their Euclidean distance based on CAGS [21].

### Clinical prognosis and molecular characterization of different CAG patterns in HCC patients

HCC patients were assigned to either a high CAGS group or a low CAGS group, based on the median CAGS value. Kaplan–Meier survival analysis was used to compare the overall survival (OS) period of the high CAGS and low CAGS HCC patients and that of three CAG patterns. The mutation annotation format (MAF) files of the HCC patients were acquired from TCGA, and the somatic mutation landscapes of the three CAG patterns were compared. The immune scores, stromal scores, and tumor purity of the HCC patients were calculated using the Estimation of STromal and Immune cells in MAlignant Tumor tissue using the Expression data (ESTIMATE) algorithm [22, 23]. The infiltration levels of 22 immune cells were quantified in each HCC patient using the Cell type Identification by Estimating Relative Subsets of RNA Transcripts (CIBERSORT) algorithm [24, 25, 26, 27, 28]. The tumor microenvironment (TME) of different CAG patterns was compared using either the Wilcoxon test or the ANOVA. The metabolism‐related signatures in each HCC patient were quantified by PCA, and the metabolic discrepancies of the three CAG patterns were compared in a heatmap.

### Prediction of ICB treatment response

To detect the potential ability of the CAGS in predicting ICB treatment response, we downloaded the expression data and clinical features of two ICB cohorts (i.e., the IMvigor210 urothelial cancer [29] cohort and the GSE78220 melanoma [30] cohort). The IMvigor210 and GSE78220 expression matrices were transformed into TPM data. Two patients in the GSE78220 cohort were excluded because they lacked survival data or they had previously received any other on‐therapy. For the duplicated probes in each matrix, only the mean expression values were preserved. The CAGS of each patient was quantified and was used to predict the OS condition and the ICB therapeutic response of the patients, which included progress disease (PD), stable disease (SD), partial remission (PR), and complete remission (CR).

### In‐house immunohistochemistry (IHC)

HCC tissue microarrays (TMA) LVC1505 and LVC1531 were purchased from Fanpu Biotechnology Co., Ltd, Guilin, China (http://www.fanpu.com/). All the tumor specimens were pathologically diagnosed with primary HCC, and the patients did not receive drug interventions before surgery. IHC staining was performed using the anti‐STAG1 antibody (Abcam Co., Ltd, ab246988) according to the procedure previously reported [31, 32, 33, 34]. The dilution of the anti‐STAG1 antibody was 1 : 100. The patients provided written informed consent for the use of their tissue samples. This study has been approved by the Ethics Committee of The First Affiliated Hospital of Guangxi Medical University [No.2022‐KY‐E‐017]. The protocols conformed to the guidelines set by the Declaration of Helsinki.

### Global HCC microarrays and RNA sequencing (RNA‐seq) datasets

Public HCC datasets, including microarrays and RNA‐seq matrices, were searched in the Gene Expression Omnibus (GEO) and ArrayExpress databases. The query keywords were as follows: HCC OR hepatocellular carcinoma OR liver cancer OR liver tumor. The inclusion criteria were as follows: (a) The specimens should be primary HCC tissue samples from humans, (b) the patients did not receive preoperative treatment, and (c) the sample size of each platform should be ≥ 6. TCGA was combined with the Genotype‐Tissue Expression (GTEx) project. We used the combat function to remove the batch effect of the same GEO platform and that of the integrated TCGA‐GTEx dataset. Data normalization was performed with the aid of the limma‐voom (The Walter and Eliza Hall Institute of Medical Research, Melbourne, Australia) package in r v3.6.1.

### mRNA expression statuses of CAG and HCC differentially expressed genes (DEGs) based on global HCC data

The mRNA expression level of STAG1 and 16 other CAGs was calculated using the enrolled global HCC datasets. Standard mean difference (SMD) was chosen as a comprehensive index for appraising the relative expression status of CAG, where an SMD score > 0 or < 0 (*P* value < 0.05) represented an upregulated or downregulated trend, respectively. Kaplan–Meier OS analysis was performed to evaluate the prognostic value of STAG1 mRNA in the TCGA‐HCC cohort.

### STAG1 co‐expression genes (CEGs)

STAG1 CEGs were identified from the processed global HCC datasets by calculating Pearson correlation coefficients, where coefficients ≤ −0.3 or ≥ 0.3 and *P* values < 0.05 represented negatively correlated STAG1 CEGs or positively correlated STAG1 CEGs, respectively. Weighted gene co‐expression network analysis (WGCNA) was conducted to determine the high‐CAGS HCC phenotype‐related module genes.

### Multiscale embedded gene co‐expression network analysis (MEGENA)

We acquired the HCC differentially expressed STAG1 CEGs by intersecting the upregulated DEGs with the positively correlated STAG1 CEGs, and by overlapping the downregulated DEGs with the negatively correlated STAG1 CEGs. The functional networks of the HCC differentially expressed STAG1 CEGs were identified by analyzing their topological structure. Metascape was utilized to annotate the biological functions of the HCC differentially expressed STAG1 CEGs.

### Putative transcriptional targets of STAG1 based on global chromatin immunoprecipitation sequencing (ChIP‐seq) data

A total of 58 Cistrome ChIP‐seq datasets were utilized to identify the candidate transcriptional target genes of the STAG1 factor. The cutoff value for filtering the putative targets of STAG1 was set at a score ≥ 3.0. The transcriptional regulatory relationships were further validated by peak annotations and Pearson co‐expression analysis.

### Potential therapeutic agents for HCC

The CellMiner database is a repository of molecular and pharmacological data based on 60 cell lines from the National Cancer Institute (NCI‐60), which contains the transcriptome data of 60 cancer cell lines and over 100,000 natural products and chemical compounds [35]. Herein, both US Food and Drug Administration (FDA) approved drugs, and clinical trial drugs were selected for in‐depth analysis. To screen for putative anticancer therapeutic agents, we first analyzed the discrepancies of half maximal inhibitory concentration (IC_50_) in the STAG1 CEG high and low expression groups by targeting the STAG1‐enriched molecular pathways. We computed Pearson correlation coefficients between the IC_50_ values of the screened agents and the STAG1 CEG expressions. Computer‐aided drug‐protein molecular docking was conducted to fundamentally validate the potential therapeutic agents for treating HCC.

## Results

### Distinct CAG patterns in HCC patients with different clinical phenotypes

**Fig. 1:**
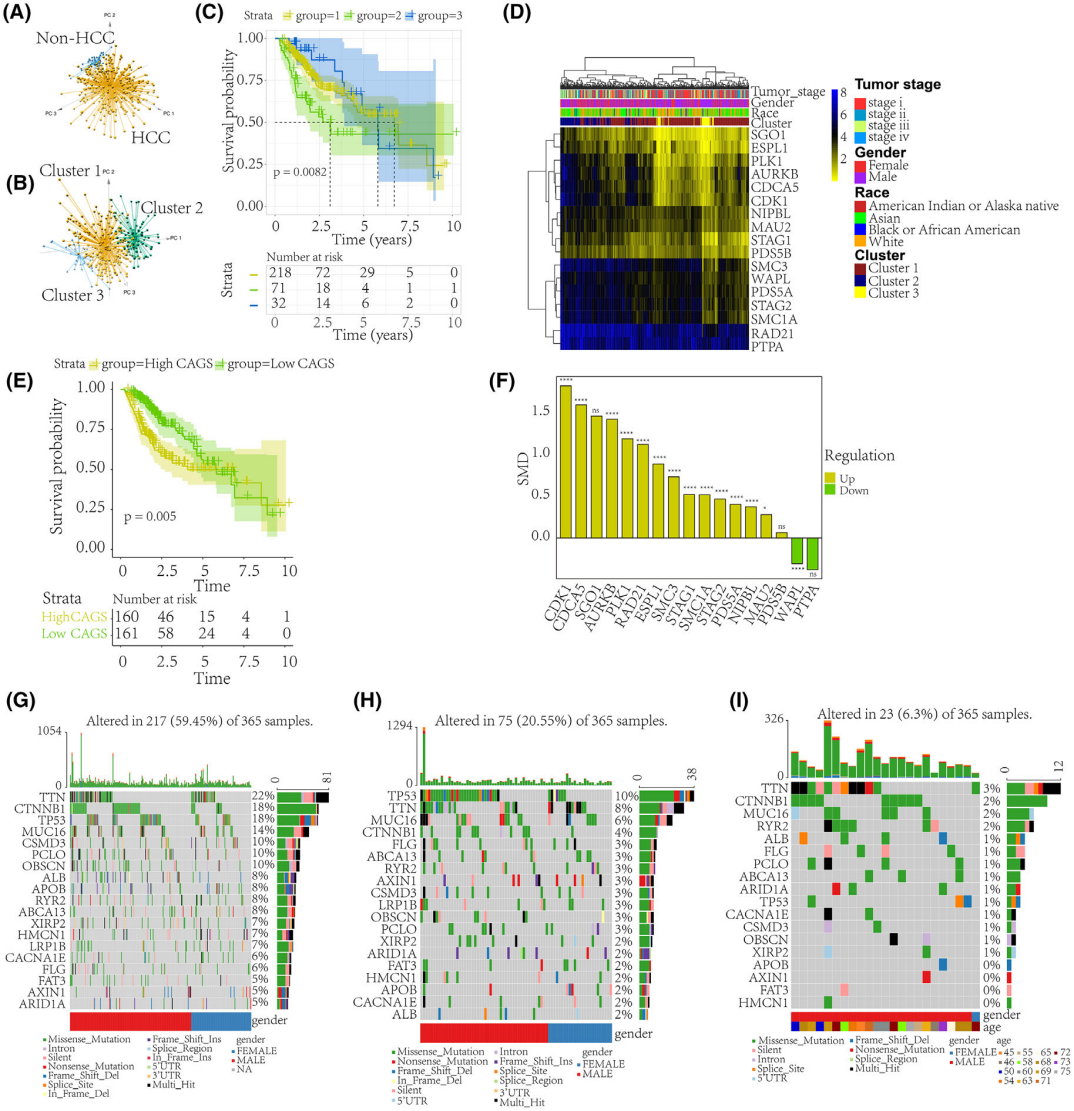
STAG1‐mediated distinct prognostic and mutation phenotypes in HCC. Given that STAG1 is a pivotal component of cohesin, it and 16 other CAGs were collected to explore their biological functions. (A) Using STAG1 and 16 other CAGs, HCC tissue samples could be preferably differentiated from adjacent normal liver tissue samples. (B) HCC patients were assigned to three clusters based on their cohesin‐associated signatures. (C) HCC patients classified into cluster 2 were found to have a worse survival outcome than the patients in the other clusters. A log‐rank test was used to identify the survival discrepancy. (D) The clinical characteristics of different HCC clusters. (E) The CAG signatures in each patient were quantified using single‐sample gene set enrichment analysis. The calculated values, termed CAGS, were used to classify the HCC patients. The low CAGS HCC patients displayed a significant survival advantage. A log‐rank test was used to identify the survival discrepancy. (F) Dysregulated expression levels of STAG1 and 16 other CAGs in the global HCC data (sample size: T_*AURKB*_ = 3831, N_*AURKB*_ = 3102; T_*CDCA5*_ = 3412, N_*CDCA5*_ = 2653; T_*CDK1*_ = 2347, N_*CDK1*_ = 1832; T_*ESPL1*_ = 3926, N_*ESPL1*_ = 3428; T_*MAU2*_ = 2141, N_*MAU2*_ = 1706; T_*NIPBL*_ = 3334, N_*NIPBL*_ = 2729; T_*PDS5A*_ = 3081, N_*PDS5A*_ = 2481; T_*PDS5B*_ = 3055, N_*PDS5B*_ = 2451; T_*PLK1*_ = 3414, N_*PLK1*_ = 3036; T_*PTPA*_ = 384, N_*PTPA*_ = 305; T_*RAD21*_ = 3395, N_*RAD21*_ = 3023; T_*SGO1*_ = 397, N_*SGO1*_ = 296; T_*SMC1A*_ = 3386, N_*SMC1A*_ = 3008; T_*SMC3*_ = 3306, N_*SMC3*_ = 2701; T_*STAG1*_ = 3313, N_*STAG1*_ = 2692; T_*STAG2*_ = 3414, N_*STAG2*_ = 3036; T_*WAPL*_ = 425, N_*WAPL*_ = 340, where T and N stand for HCC and non‐HCC group, respectively). Distinct mutation phenotypes of the three HCC clusters are shown in G–I, where *TP53* is identified as the most frequently mutated gene in the HCC patients of cluster 2. CAG, cohesin‐associated gene; CAGS, cohesin‐associated gene scores; HCC, hepatocellular carcinoma; STAG1, stromal antigen 1. ****P value < 0.0001; *P value < 0.05; ns, not significant.

In this study, the CAGs showed a highly effective performance in differentiating HCC tissue samples from adjacent normal liver tissue samples (Fig. 1A). A total of three CAG patterns were identified from the HCC patients (Fig. 1B), where cluster 2 showed a worse OS outcome than any other clusters (Fig. 1C). Figure 1D summarizes the clinical characteristics of the different HCC clusters. We also quantified the CAGS of each HCC patient, and noticed that the low CAGS HCC patients displayed a significant survival advantage (Fig. 1E). Among the 17 CAGs, a total of 13 signatures were significantly overexpressed in global HCC tissue samples (Fig. 1F).

### Metabolic characterization and genetic alterations and TME landscapes of three CAG patterns

**Fig. 2:**
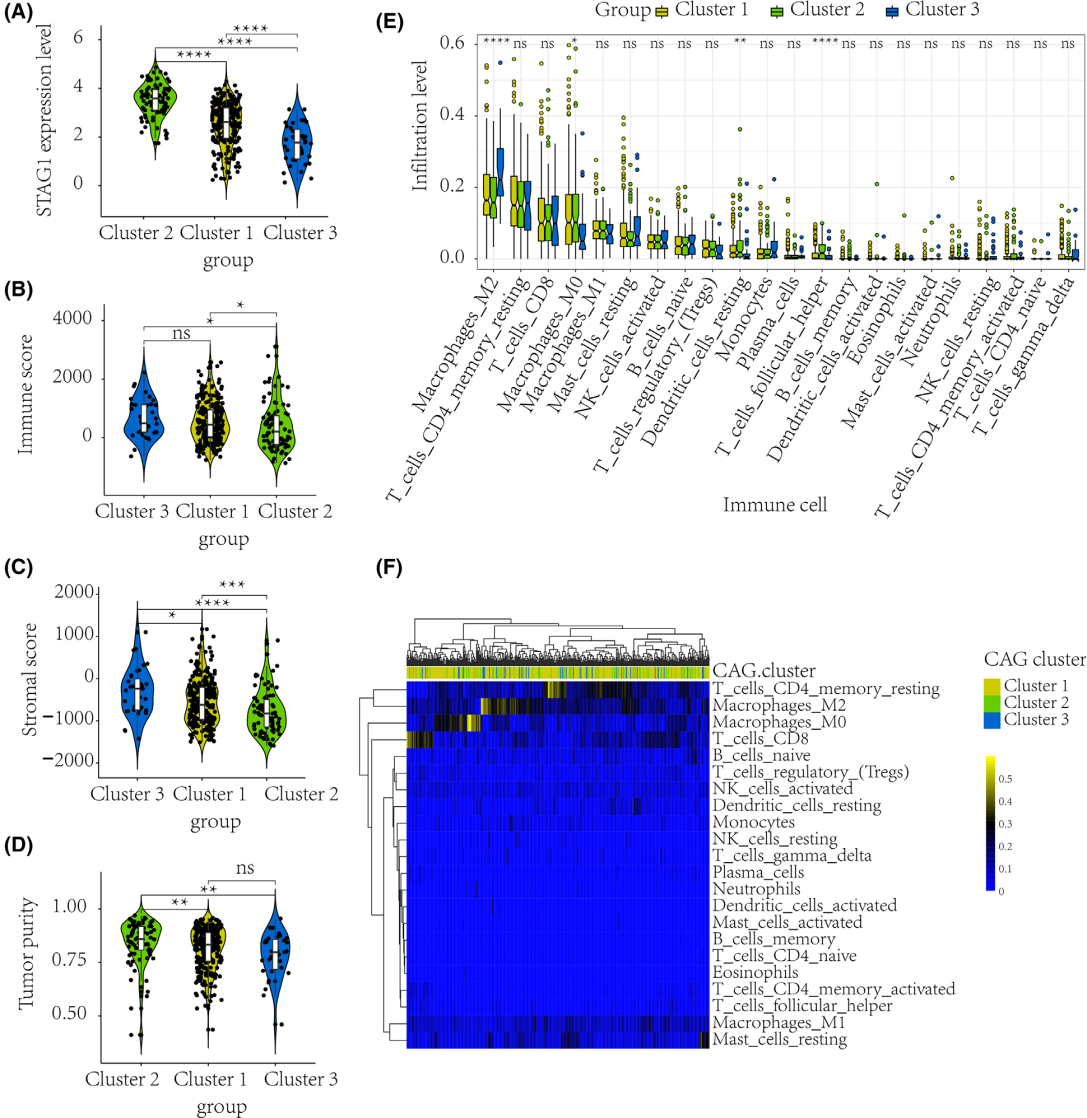
Distinct TME characterization of HCC patients. According to the identified CAG clusters, the TME characteristics of different HCC patients were compared. A Wilcoxon test was used to compare the intercluster immune and stromal scores (sample size: N_cluster1_ = 251; N_cluster2_ = 85; N_cluster3_ = 35). The STAG1 mRNA expression level was significantly higher in CAG cluster 2 (A). HCC patients in CAG cluster 2 with poor prognosis exhibited prominently lower immune scores and stromal scores (B–C), and possessed higher tumor purity (D) than the other HCC patients. An ANOVA method was used to compare the immune infiltration levels among three clusters (sample size: N_cluster1_ = 251; N_cluster2_ = 85; N_cluster3_ = 35). HCC patients in CAG cluster 2 also showed higher infiltration levels of resting dendritic and T follicular helper cells (E–F). HCC, hepatocellular carcinoma; CAG, cohesin‐associated gene; TME, tumor microenvironment. ****P value < 0.0001; ***P value < 0.001; **P value < 0.01; *P value < 0.05; ns, not significant.

Given the essence of tumor metabolic reprograming in cancer progression [36], we first compared the metabolic characterization based on the identified CAG clusters. Notably, the HCC patients in CAG cluster 2, with a poorer prognosis than any of the other clusters, displayed prominently lower metabolism levels of drug, retinol, and xenobiotics (Fig. S2A–C). Additionally, distinct gene mutation phenotypes were identified among the three HCC CAG clusters (Fig. 1G–I), where *TP53* was the most frequently mutated gene in cluster 2. Moreover, the mutation co‐occurrence between *TP53* and other genes, such as *TTN*, *MUC16*, and *CTNNB1*, was more frequent in HCC CAG cluster 2 (Fig. S2D–F) than in the other clusters. As is shown in Fig. 2A, the STAG1 mRNA expression level was compared between different CAG clusters. Pertaining to TME, HCC CAG cluster 2 exhibited significantly lower immune and stromal scores (Fig. 2B,C) but possessed higher tumor purity (Fig. 2D). Additionally, HCC CAG cluster 2 showed higher infiltration levels of resting dendritic and T follicular helper cells (Fig. 2E,F) than the other clusters.

### Potential ICB immunotherapy response prediction ability of CAGS

**Fig. 3:**
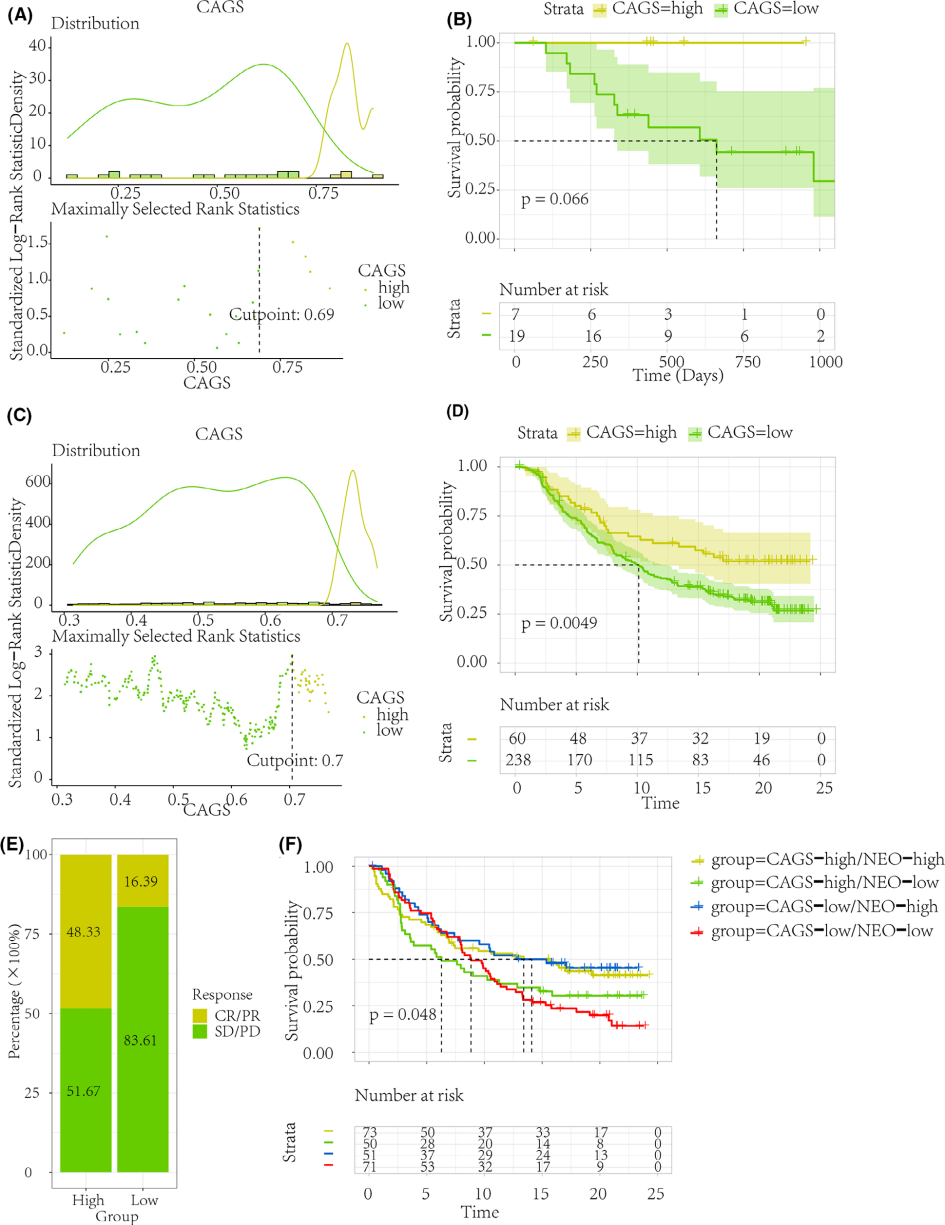
Prediction of immune checkpoint blockage therapy response. To detect the potential ability of CAGS in predicting ICB treatment response, the CAGS were quantified using IMvigor210 urothelial cancer and GSE78220 melanoma ICB cohorts. Patients with high CAGS exhibited a better overall survival condition than that with low CAGS in both the anti‐PD‐1 (GSE78220, A–B) and anti‐PD‐L1 (IMvigor210, C–D) ICB therapy cohorts. (E) The high CAGS group had a higher responsive rate (response/nonresponse = 48.33%/51.67%) to ICB treatment than the low CAGS group (response/nonresponse = 16.39%/83.61%). (F) Overall survival analysis of ICB therapy cohorts based on both CAGS and NEO status. CAGS, cohesin‐associated gene scores; ICB, immune checkpoint blockage; NEO, neoantigen burden.

As is shown in Fig. S3, higher STAG1 expression level predicted better OS outcomes in patients who received immunotherapy. In light of the role of CAGS in predicting OS for HCC patients, we assumed that CAGS may be helpful in predicting the response to ICB immunotherapy. After categorizing the cancer patients based on the optimal cutoff value of CAGS, those with a high CAGS exhibited a clinical benefit in both the anti‐PD‐1 (GSE78220, Fig. 3A,B) and anti‐PD‐L1 (IMvigor210, Fig. 3C,D) ICB therapy cohorts. Furthermore, as seen in Fig. 3E, the high CAGS group had a higher responsive rate (response/nonresponse = 48.33%/51.67%) to ICB treatment than the low CAGS group (response/nonresponse = 16.39%/83.61%). However, with the combination of CAGS and tumor neoantigen (NEO) burden, it was observed that the CAGS‐high/NEO‐high group had a comparable survival probability as the CAGS‐low/NEO‐high group, which suggested that the survival impact of CAGS may be inferior to that of NEO level (Fig. 3F).

### Comprehensive overexpression of STAG1 in global HCC mRNA data and in‐house IHC validation

Because STAG1 is the core subunit of the cohesin complex protein and it serves as a TF, its expression status in HCC tissue samples was further explored. A total of 37 platform matrices were enrolled in this study (Table S1), which covered 76 datasets from 12 countries (Table S2). The SMD forest plot indicated that STAG1 was upregulated in 3313 HCC tissue samples in comparison with 2692 non‐HCC tissue samples (Fig. S4A). The sensitivity analysis plot indicated that the included datasets could not explain the major source of heterogeneity (Fig. S4B). The funnel plot implied insignificant publication bias, which showed the stability of the quantitative synthesis result (Begg's test: *P* value = 0.067) (Fig. S4C). The summary characteristics operating curve showed a moderate discriminatory ability of STAG1 (Fig. S4D), with weak sensitivity (Fig. S4E) and moderate specificity (Fig. S4F). Fagan's nomogram and likelihood ratio forest plots indicated the general accuracy of STAG1 in differentiating between the HCC and non‐HCC tissue samples (Fig. S4G–I). However, the OS prediction ability of STAG1 was insignificant in HCC patients (data not shown).

**Fig. 4:**
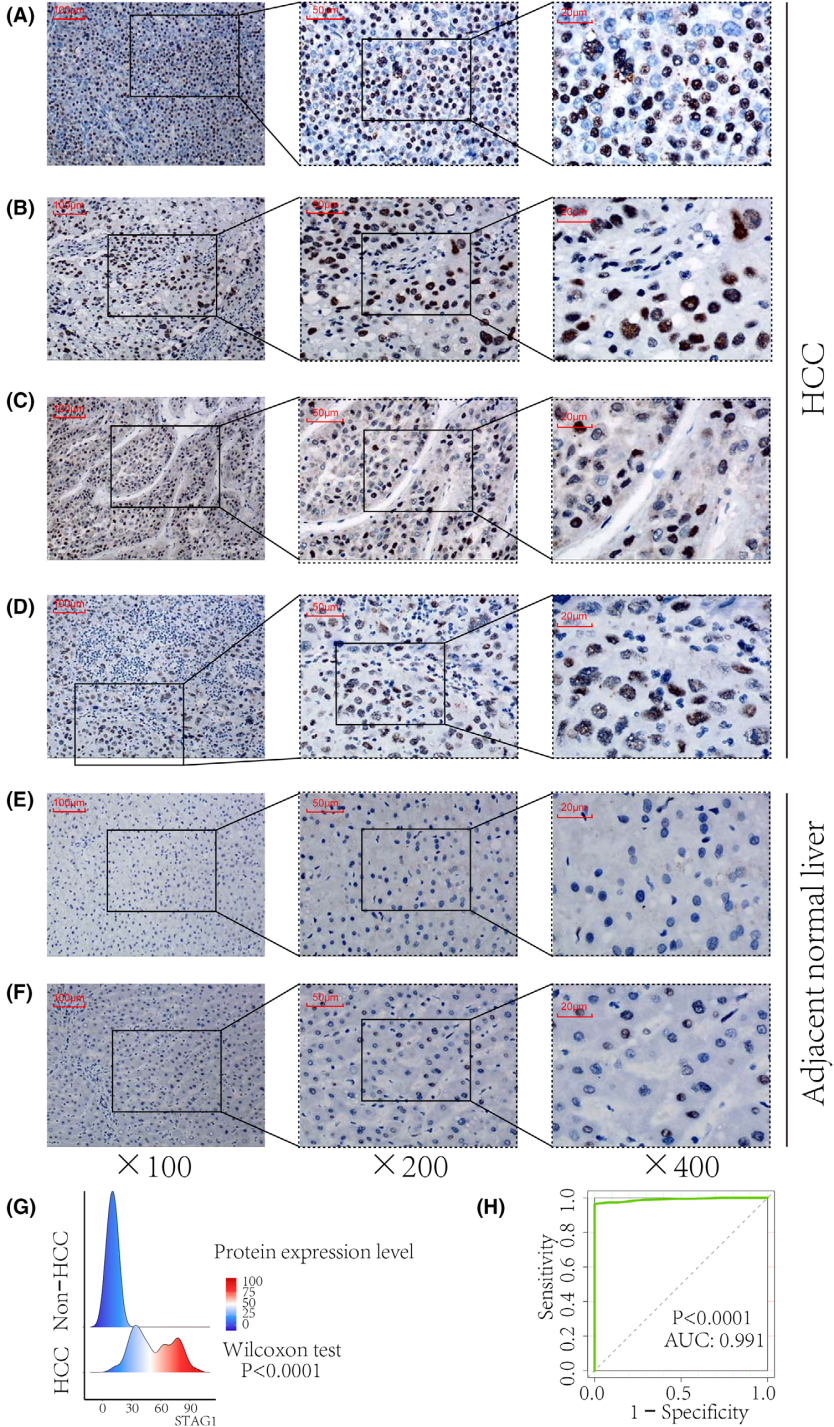
In‐house immunohistochemistry staining of STAG1 transcriptional factor in HCC tissue samples. STAG1 protein was stained in the nucleus of the HCC cells (A–D) and compared with healthy cells (E–F). Increased protein expression of STAG1 in HCC displayed a strong ability in differentiating HCC from non‐HCC tissue samples (G–H). HCC, hepatocellular carcinoma. The scale bars in panels A–F represent 100, 50, and 20 μm, from left to right.

More importantly, the STAG1 protein was remarkably stained in the nucleus of the HCC cells (Fig. 4A–D) in comparison with the non‐HCC cells (Fig. 4E,F), thus validating the overexpression trend of STAG1 in HCC tissue samples (Fig. 4G,H).

### Prospective signaling pathways and transcriptional regulation of STAG1 in HCC

**Fig. 5:**
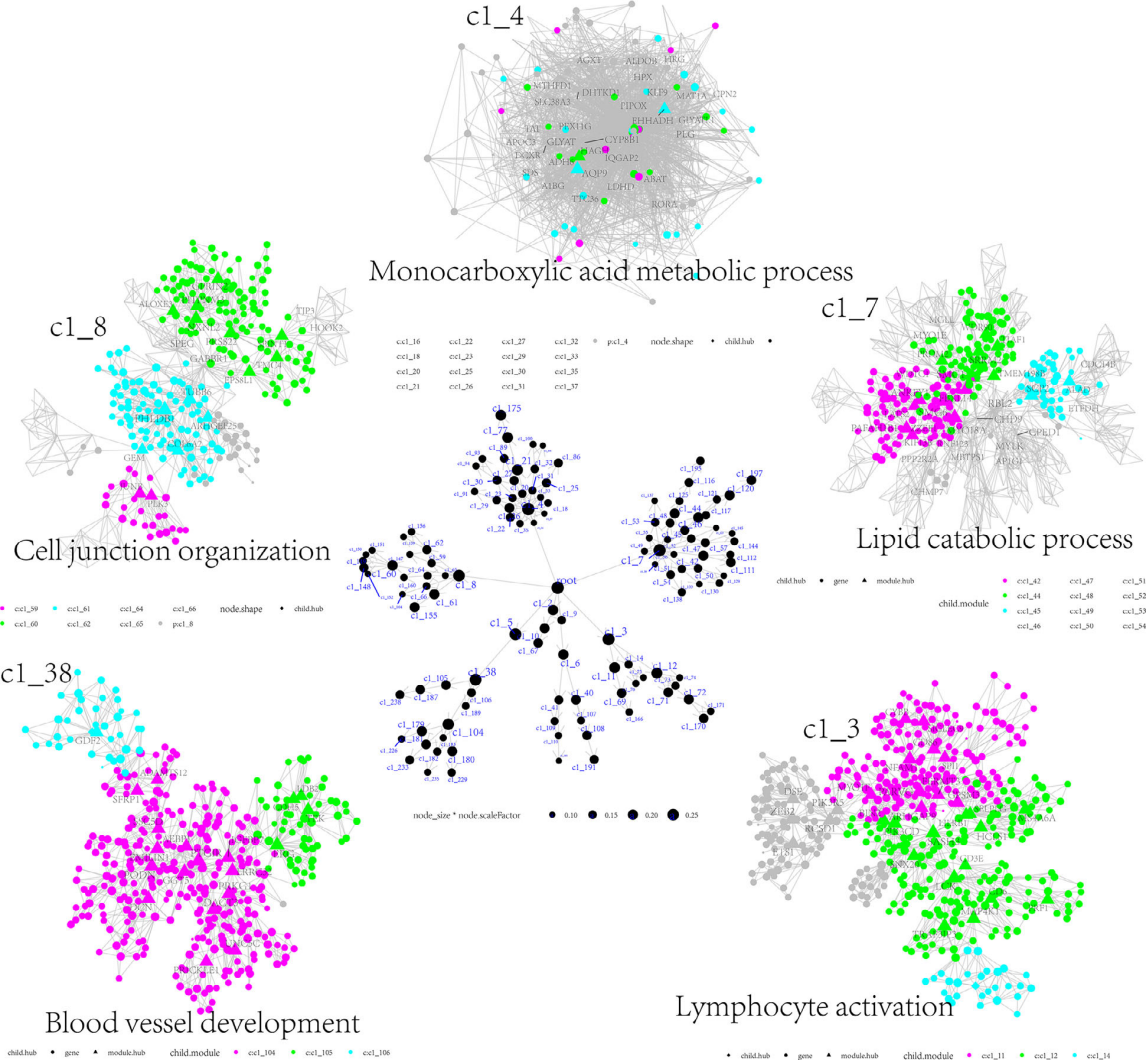
Potential functional modules of downregulated STAG1 negative co‐expression genes in HCC. Based on the downregulated STAG1 negative co‐expressed genes, a total of five predominant modules were identified using the multiscale embedded gene co‐expression network analysis (MEGENA).

**Fig. 6:**
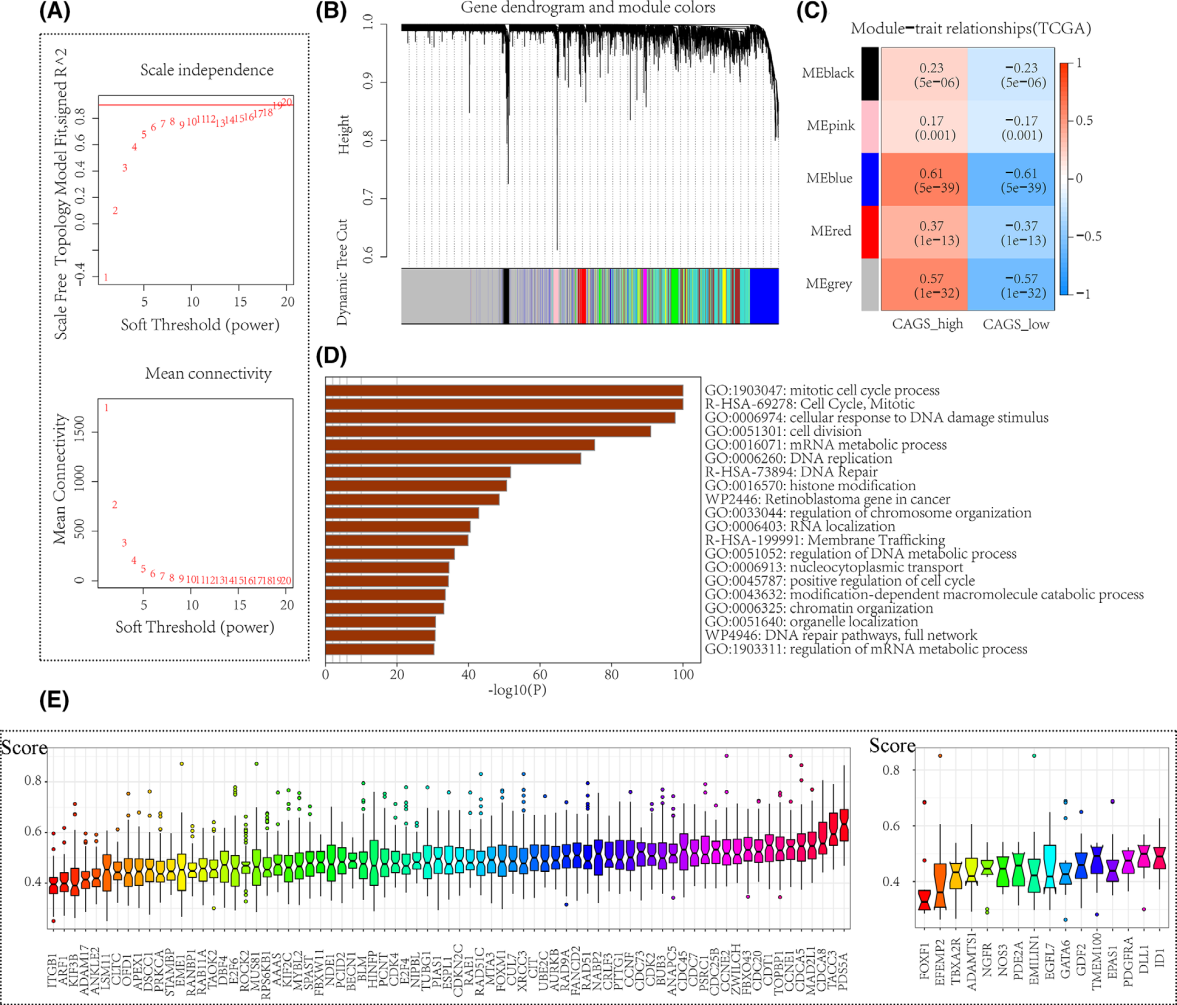
Prospective signaling pathways of upregulated STAG1 positive co‐expression genes in hepatocellular carcinoma. We performed weighted gene co‐expression network analysis (WGCNA) to determine the high‐CAGS HCC phenotype‐related module genes. (A) A soft threshold of 19 was set to construct a scale‐free topological model. (B–C). A blue gene module exhibited a highly positive correlation with the CAGS‐high phenotype in HCC. (D) The blue gene module was predominantly enriched in the mitotic cell cycle process. (E) The similarity scores of the STAG1 putative transcriptional targets were calculated, and *PDS5A* and *ID1* were identified as the core genes in blood vessel development and mitotic cell cycle process, respectively. CAGS, cohesin‐associated genes scores; HCC, hepatocellular carcinoma.

**Fig. 7:**
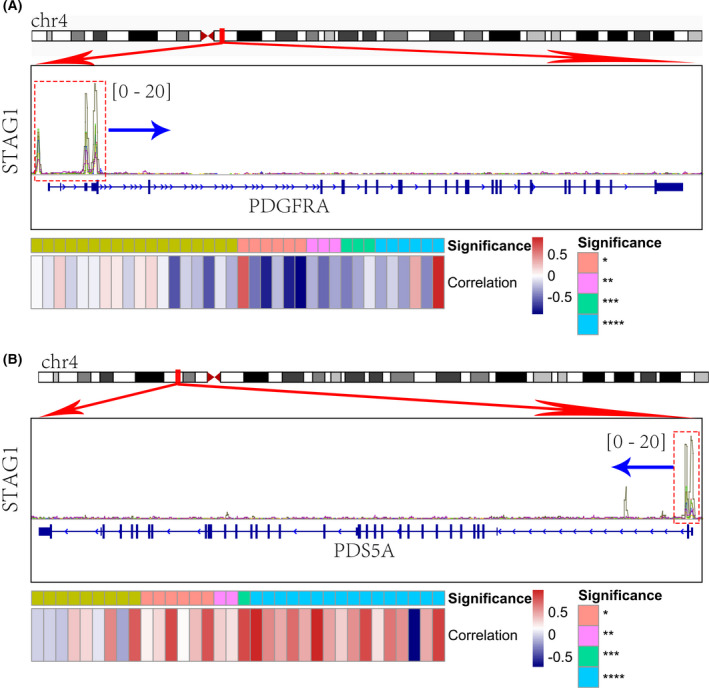
Transcriptional regulation of STAG1 in mitotic cell cycle process and blood vessel development. We identified two transcriptional targets of STAG1 in hepatocellular carcinoma. (A) *PDS5A* in the mitotic cell cycle process. (B) *PDGFRA* in blood vessel development.

By adopting a network embedding algorithm, a total of five predominant functional modules were identified from the downregulated STAG1 negative CEGs (Fig. 5), which were enriched in lymphocyte activation (c1_3), monocarboxylic acid metabolic process (c1_4), lipid catabolic process (c1_7), cell junction organization (c1_8), and blood vessel development (c1_38). Additionally, we constructed a scale‐free co‐expression network based on the upregulated STAG1‐positive CEGs (soft threshold = 19) (Fig. 6A). The blue module, defined as the high‐CAGS HCC phenotype‐related module (Fig. 6B,C), was predominantly enriched in the mitotic cell cycle process (Fig. 6D). The similarity scores of the STAG1 putative transcriptional targets were calculated, and PDS5 cohesin‐associated factor A (*PDS5A*) and inhibitor of DNA binding 1, HLH protein (ID1) were identified as the core genes in the mitotic cell cycle process and blood vessel development, respectively (Fig. 6E). More importantly, the downregulated platelet‐derived growth factor receptor alpha (PDGFRA) in blood vessel development (Fig. 7A) and upregulated *PDS5A* in the mitotic cell cycle process (Fig. 7B) were predicted as two transcriptional targets of STAG1.

As is shown in Fig. S5A, PDGFRA mRNA was significantly downregulated in 3348 HCC when compared with 2954 normal liver tissue specimens. Decreased PDGFRA showed a moderate discriminatory ability between HCC and normal liver tissue (Fig. S5B,C). The STAG1 expression level was inversely correlated with the mRNA level of PDGFRA (Fig. S6). Based on the potential role that STAG1 has on blood vessel development in HCC, we subsequently explored whether STAG1 could transcriptionally regulate the transcription of vascular endothelial growth factor A (VEGFA). Intriguingly, STAG1 showed an obvious transcriptional factor binding intensity in the promoter region of VEGFA (Fig. S7A). Furthermore, STAG1 was positively correlated with the expression level of VEGFA (Fig. S7B).

### Promising anti‐HCC agents by targeting STAG1 transcriptional mechanisms

**Fig. 8:**
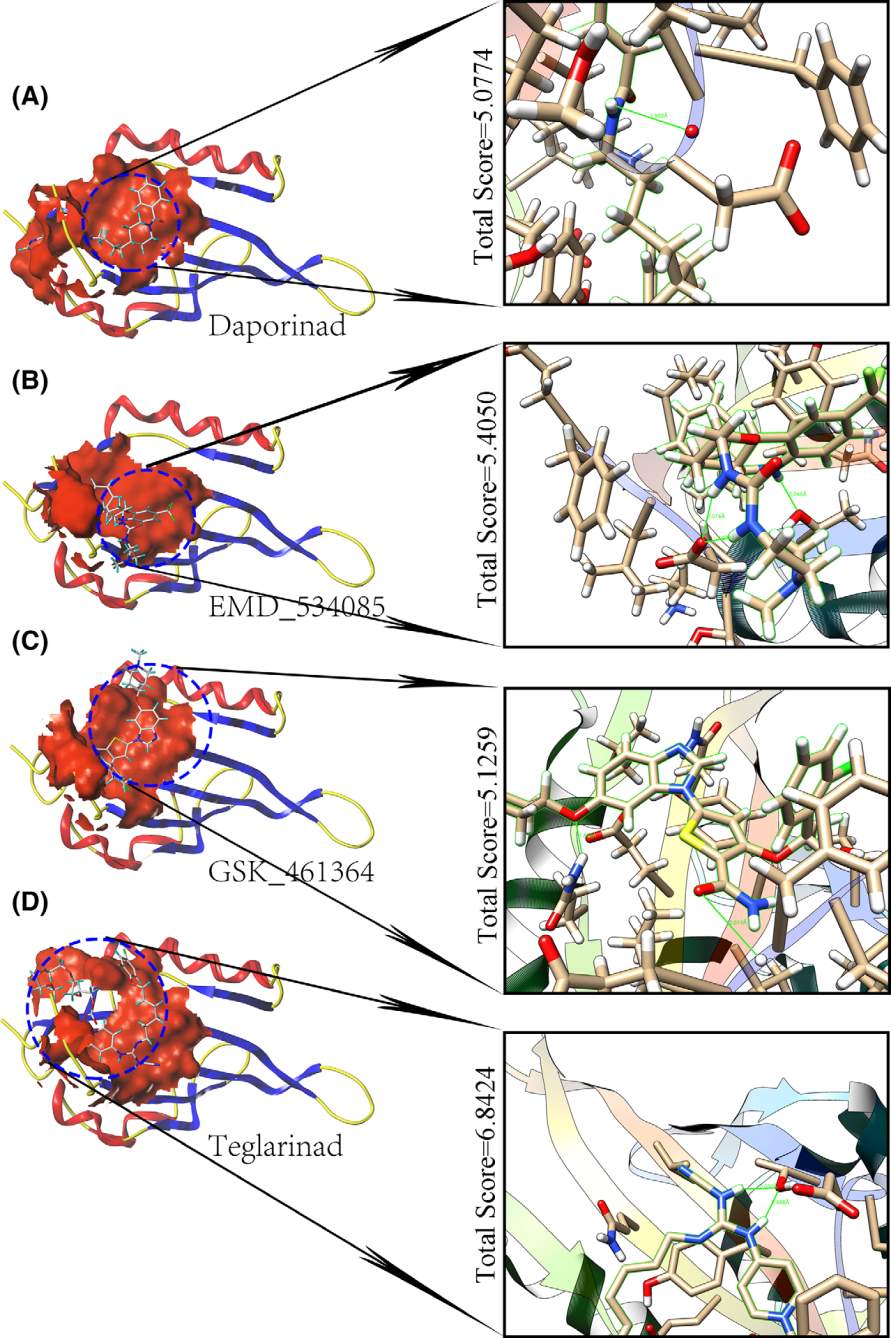
Putative therapeutic agents for treating hepatocellular carcinoma. Molecular docking between EPAS1 and four antitumor agents, (A) Daporinad (nicotinamide phosphoribosyl transferase inhibitor). (B) EMD_534085 (mitotic kinesin‐5 inhibitor). (C) GSK_461364 (polo‐like kinase 1 inhibitor). (D) Teglarinad (a prodrug of nicotinamide phosphoribosyl transferase inhibitor), which has been approved by the US Food and Drug Administration.

Based on the enriched gene ontology terms of blood vessel development and mitotic cell cycle process, a total of 91 putative transcriptional targets were inputted to screen for sensitive anti‐HCC agents, either FDA‐approved drugs or clinical trial drugs, and were used to identify the potential interventional targets for treating HCC patients. Surprisingly, downregulated endothelial PAS domain protein 1 (EPAS1) (SMD = −0.6386, *P* value < 0.0001) exhibited a significant negative correlation with the IC_50_ values of 14 agents (such as TAK‐960 analog, methotrexate, benzimate, TAK Plk inhibitor, oxaliplatin, volasertib, and teglarinad) and was positively correlated with that of XAV‐939 and AZD‐8055 (Figs S8 and S9). Among them, daporinad (a nicotinamide phosphoribosyl transferase inhibitor), EMD‐534085 (a mitotic kinesin‐5 inhibitor), GSK‐461364 (a polo‐like kinase 1 inhibitor), and teglarinad (a prodrug of nicotinamide phosphoribosyl transferase inhibitor) ligands could be docked by the EPAS1 protein with a high total score (all with total scores > 5.0). Further interaction analysis implied that the hydrogen bond was the predominant type of force (Fig. 8).

## Discussion

HCC is a lethal abdominal malignancy with few effective intervention strategies in its late stages [37, 38]. The advent of immunotherapy, represented by ICB therapy, created novel opportunities for advanced HCC patients [39, 40, 41]. In this study, we established a CAGS‐based signature scoring system and identified three CAG patterns in HCC patients, which may be used to define distinct clinical prognosis, TME, and genetic mutation characterization. Moreover, a high CAGS may be a potential indicator for therapeutic response to anti‐PD‐1/PD‐L1 ICB treatment. STAG1, as an important CAG TF, was comprehensively explored in the transcriptional regulatory mechanisms of blood vessel development and the mitotic cell cycle process in HCC, where EPAS1 may be a drug‐treatment target for HCC treatment.

To date, numerous biomarkers and models have been found to predict immunotherapy response [42, 43, 44]. The novelty of the present study is that we predicted the response status to ICB therapy by converting 17 CAG signatures into personalized ssGSEA scores [45], rather than by directly depending on the expression values. Intriguingly, a high CAGS predicted poor OS prognosis in HCC patients but exhibited a clinical advantage in anti‐PD‐1/PD‐L1 immunotherapy, with a higher response rate in comparison with the low CAGS group (response/nonresponse = 48.33%/51.67%). Additionally, a combination of high NEO burden [46] and high CAGS may be used to predict a better clinical prognosis when receiving standard ICB treatment.

It is well‐known that TME plays a pivotal role in promoting the invasion and metastasis of HCC by regulating immune and stromal cells [47, 48]. In this study, we identified three CAGS patterns from the HCC patients and revealed the distinct clinical and molecular characteristics among them. We noticed that the CAGS cluster 2 group displayed an attenuated toxic metabolism level and had an increased mutation rate of *TP53*. Further exploration revealed that the CAGS cluster 2 was characterized by high tumor purity along with deceased immune and stromal scores. To the best of our knowledge, cohesin mutations are commonly detected in a range of malignancies [49, 50, 51], and the *TP53* suppressor gene is one of the most common mutation genes in HCC [52]. More importantly, there are intimate associations between cohesin and *TP53* mutations. For instance, there is a high co‐occurrence between *TP53* gene alterations and cohesin mutations [53]. The STAG1 cohesin subunit has been reported to serve as a direct transcriptional target of *TP53* [54]. In response to genotoxic stresses, SATG1 mRNA expression could be induced in a *TP53*‐dependent manner, and endogenous STAG1 protein expression increased significantly when exposed to ultraviolet radiation [54]. Notably, STAG1 also participated in DNA damage repair [55], in addition to the renowned genome guardian, *TP53* [56]. Moreover, *TP53* gene mutation abrogated its role in promoting DNA damage repair and cellular apoptosis, thus leading to severe cell carcinogenesis [57]. In this setting, CAGs may be used to define a poorer HCC phenotype with higher tumor purity and more *TP53* mutations.

Furthermore, the present study shed light on the transcriptional mechanisms of the STAG1 cohesin subunit involving blood vessel development and the mitotic cell cycle process in HCC. We first confirmed the overexpression of STAG1 in 3313 HCC tissue samples based on multicentered HCC bulk RNA‐seq data (SMD = 0.54). Intriguingly, it was observed that the expression level of STAG1 was increased after hsa‐miR‐23a or hsa‐miR‐27a knockout [58], which suggested that microRNA was involved in the overexpression of STAG1 in HCC. Furthermore, according to six ChIP‐seq datasets [59, 60, 61], the *PDS5A* and *PDGFRA* promoter regions both exhibited remarkably high TF‐DNA binding intensities of STAG1 (range: 0–20). Based on the global HCC mRNA expression data from 12 ethnicities, *PDS5A* was an upregulated gene and positively correlated with STAG1; conversely, *PDGFRA* was downregulated and negatively correlated with STAG1.

Similar to the cohesin core subunit STAG1, the *PDS5A* regulatory subunit is mainly involved in modulating sister chromatid cohesion during mitosis [62]. The PDS5A protein participates in the dynamic association between cohesin and chromatin and protects the replication forks from degradation [63]. After the deletion of the PDS5 protein, cell cycle progression was inhibited [64]. In this context, STAG1 TF is proposed to positively regulate cell cycling by targeting *PDS5A*, thus promoting mitotic division and malignant proliferation of HCC cells. Additionally, STAG1 was correlated with vascular development. It was demonstrated that STAG1 and STAG2 balanced the production of hematopoietic/vascular progenitors in a *Zebrafish* embryo model [65]. In the shTMEM30A‐treated primary human retinal endothelial cells, STAG1 was significantly downregulated and the vascular formation was inhibited [66]. Herein, we proposed that *PDGFRA* may be a transcriptional target of STAG1 in HCC. *PDGFRA* encodes a tyrosine kinase receptor and is involved in embryonic development and wound healing [67]. The *PDGFRA* signaling pathway may promote angiogenesis, carcinogenesis, and tumor dissemination [68, 69]. Moreover, *PDGFRA* was downregulated in the HCC tissue samples but overexpressed in endothelial cells, and it correlated with vascular invasion and forecast poorer OS and disease‐free survival in HCC patients [70]. Taken together, these studies showed that downregulated *PDGFRA* may be negatively regulated by STAG1 TF in HCC cells but positively correlated with the emerging vessels surrounding the tumor lesion. To support our hypothesis, we also investigated the transcriptional activity of STAG1 and the tumor angiogenesis promoting factor, VEGFA. Surprisingly, STAG1 was positively correlated with VEGFA and exhibited a specific transcriptional factor binding peak at the upstream promoter region of the *VEGFA* gene. It was conceivable that STAG1 may promote the angiogenesis in HCC by positively regulating the transcription of VEGFA. The present study revealed two important transcriptional targets for STAG1 in HCC development. In the future, *in vitro* and *in vivo* assays must be conducted to validate the transcriptional mechanism of STAG1 underlying HCC.

Several anti‐HCC agents were also identified by targeting the EPAS1 protein, all with high total scores. Interestingly, a previous study had reported that microRNA‐3609 could attenuate the expression of EPAS1 and sensitize HCC cells to sorafenib treatment [71]; that study was the first to reveal the potential role of EPAS1 in HCC treatment. The present study further expanded on that finding by identifying EPAS1 as the targetable protein in treating HCC. Specifically, teglarinad (GMX1777) exhibited the highest total score when docked by the EPAS1 protein, and it was found that higher EPAS1 expression correlated with higher sensitivity of teglarinad to treat HCC cells. Teglarinad is believed to suppress the development of tumor cells by interfering with DNA repair, abrogating angiogenesis, and enhancing radiation efficacy [72]. However, it is necessary to verify the potential implications of the screened anti‐HCC agents at the cell and animal levels, or even at the clinical trial level. It is believed that the development of emerging technologies of in‐silico drug designs and artificial intelligence will result in more effective therapies for curing HCC patients in the future.

## Conclusions

In this study, we identified three distinct CAG phenotypes from HCC patients, and a high CAGS predicted poor prognosis of HCC patients but correlated with potential clinical advantages in ICB immunotherapy. STAG1 may fuel HCC deterioration by transcriptionally regulating blood vessel development and the mitotic cell cycle.

## Conflict of interest

The authors declare no conflict of interest.

## Author contributions

(I) Study design, experimental supervision, and manuscript editing: C‐ZL, GC, R‐QH, Z‐GH, J‐JL, X‐FD, and X‐PL; (II) Experimental operation, data analysis and interpretation, and manuscript writing: C‐ZL, J‐DL, and RL; (III) Final approval of manuscript: All authors.

## Supporting information


**Fig. S1.** Design route of this research.
**Fig. S2.** Metabolic characterization and mutation co‐occurrence statuses of HCC patients. Based on the identified CAG clusters, the metabolic characterization and mutation co‐occurrence statuses of different HCC patients were compared. (A–C) The HCC patients in CAG cluster 2 with poor prognosis displayed prominently lower metabolism levels of drug, retinol, and xenobiotics than the HCC patients in the other CAG clusters. Panels D–F show the mutation co‐occurrence statuses of different CAG patterns. *TP53* mutation co‐occurrence was more frequent in the HCC patients in the CAG cluster 2 than in the patients in the other CAG clusters. Abbreviations: HCC, hepatocellular carcinoma; CAG, cohesin‐associated gene.
**Fig. S3.** Prognostic prediction ability of STAG1 in patient receiving immunotherapy. We appraised the prognostic value of STAG1 in the IMvigor210 urothelial cancer (A) and GSE78220 melanoma (B) immunotherapy cohorts. Higher STAG1 expression level predicted better overall survival outcomes in patients who received immunotherapy.
**Fig. S4.** Upregulation of STAG1 based on global HCC data. A total of 37 platform matrices were enrolled to analyze the overall expression status of STAG1 in HCC. (A) The standard mean difference forest plot indicated that STAG1 was upregulated in 3313 HCC tissue samples in comparison to 2692 non‐HCC tissue samples. (B) The sensitivity analysis plot indicated that the included datasets could not explain the major source of heterogeneity. (C) The funnel plot implied insignificant publication bias, which showed the stability of the quantitative synthesis result (Begg's test: *P* value = 0.067). (D) The summary characteristics operating curve showed a moderate discriminatory ability of STAG1, with weak sensitivity (E) and moderate specificity (F). (G–I) Fagan's nomogram and likelihood ratio forest plots indicated the general accuracy of STAG1 in differentiating between HCC and non‐HCC tissue samples. Abbreviation: HCC, hepatocellular carcinoma.
**Fig. S5.** Comprehensive mRNA expression level of PDGFRA in the HCC tissue samples. PDGFRA was significantly downregulated in the HCC tissue samples when compared with normal liver tissue specimens (A). Downregulated PDGFRA mRNA showed a moderate discriminatory ability between HCC and normal liver tissue samples (B, C). Abbreviation: HCC, hepatocellular carcinoma.
**Fig. S6.** Negative correlations between STAG1 factor and PDGFRA target. We computed the Pearson correlation coefficients between STAG1 expression level and PDGFRA expression level. STAG1 expression level was inversely correlated to PDGFRA mRNA level.
**Fig. S7.** Potential correlations between STAG1 factor and the VEGFA mRNA expression. Chromatin immunoprecipitation sequencing data were reanalyzed to explore the potential regulation between STAG1 and vascular endothelial growth factor A (VEGFA). STAG1 showed an obvious transcriptional factor binding intensity in the promoter region of VEGFA (A). STAG1 was positively correlated to the expression level of VEGFA (B).
**Fig. S8.** Potential correlations between EPAS1 expression and the sensitivity of anti‐cancer agents. We computed Pearson correlation coefficients between EPAS1 expression and half maximal inhibitory concentration of the screened agents.
**Fig. S9.** The discrepancies of half maximal inhibitory concentration in the EPAS1‐high expression group and the EPAS1‐low expression group. Note: A Wilcoxon test was conducted to compare the discrepancies of half maximal inhibitory concentration between EPAS1‐high expression group (n = 30) and the EPAS1‐low expression group (n = 30). ***P value <0.001.
**Table S1.** Overexpressed STAG1 in 37 hepatocellular carcinoma platform matrices.
**Table S2.** Fundamental dataset information of the included global hepatocellular carcinoma datasets.

## Data Availability

Datasets used in the present study are publicly available, as is listed in Table S2.

## References

[1] Sung H , Ferlay J , Siegel RL , Laversanne M , Soerjomataram I , Jemal A , et al. Global cancer statistics 2020: GLOBOCAN estimates of incidence and mortality worldwide for 36 cancers in 185 countries. CA Cancer J Clin. 2021;71:209–49. 33538338 10.3322/caac.21660

[2] Huang DQ , El‐Serag HB , Loomba R . Global epidemiology of NAFLD‐related HCC: trends, predictions, risk factors and prevention. Nat Rev Gastroenterol Hepatol. 2021;18:223–38. 33349658 10.1038/s41575-020-00381-6 PMC8016738

[3] Nault JC , Villanueva A . Biomarkers for hepatobiliary cancers. Hepatology (Baltimore, Md). 2021;73(Suppl 1):115–27. 10.1002/hep.31175 32045030

[4] Gordan JD , Kennedy EB , Abou‐Alfa GK , Beg MS , Brower ST , Gade TP , et al. Systemic therapy for advanced hepatocellular carcinoma: ASCO guideline. J Clin Oncol. 2020;38:4317–45. 33197225 10.1200/JCO.20.02672

[5] Qi Y , Fan L , Ran D , Xu J , Wang Y , Wu J , et al. Main risk factors of type 2 diabetes mellitus with nonalcoholic fatty liver disease and hepatocellular carcinoma. J Oncol. 2021;2021:7764817. 34691178 10.1155/2021/7764817 PMC8528616

[6] Pérez‐Romasanta LA , González‐Del Portillo E , Rodríguez‐Gutiérrez A , Matías‐Pérez Á . Stereotactic radiotherapy for hepatocellular carcinoma, radiosensitization strategies and radiation‐immunotherapy combination. Cancers (Basel). 2021;13:192. 10.3390/cancers13020192 PMC7825787 33430362

[7] Atwa SM , Odenthal M , El Tayebi HM . Genetic heterogeneity, therapeutic hurdle confronting sorafenib and immune checkpoint inhibitors in hepatocellular carcinoma. Cancers (Basel). 2021;13:4343. 34503153 10.3390/cancers13174343 PMC8430643

[8] Busche S , John K , Wandrer F , Vondran FWR , Lehmann U , Wedemeyer H , et al. BH3‐only protein expression determines hepatocellular carcinoma response to sorafenib‐based treatment. Cell Death Dis. 2021;12:736. 34312366 10.1038/s41419-021-04020-z PMC8313681

[9] Pan D , Hu AY , Antonia SJ , Li CY . A gene mutation signature predicting immunotherapy benefits in patients with NSCLC. J Thorac Oncol. 2021;16:419–27. 33307194 10.1016/j.jtho.2020.11.021 PMC7920921

[10] Casak SJ , Donoghue M , Fashoyin‐Aje L , Jiang X , Rodriguez L , Shen YL , et al. FDA approval summary: atezolizumab plus bevacizumab for the treatment of patients with advanced unresectable or metastatic hepatocellular carcinoma. Clin Cancer Res. 2021;27:1836–41. 33139264 10.1158/1078-0432.CCR-20-3407

[11] Wei S , Dai M , Zhang C , Teng K , Wang F , Li H , et al. KIF2C: a novel link between Wnt/β‐catenin and mTORC1 signaling in the pathogenesis of hepatocellular carcinoma. Protein Cell. 2021;12:788–809. 32748349 10.1007/s13238-020-00766-y PMC8464548

[12] França JA , Diniz MG , Bernardes VF , Costa‐Silva RC , Souza RP , Gomez RS , et al. Cohesin subunits, STAG1 and STAG2, and cohesin regulatory factor, PDS5b, in oral squamous cells carcinomas. J Oral Pathol Med. 2017;46:188–93. 27341316 10.1111/jop.12474

[13] Tothova Z , Valton AL , Gorelov RA , Vallurupalli M , Krill‐Burger JM , Holmes A , et al. Cohesin mutations alter DNA damage repair and chromatin structure and create therapeutic vulnerabilities in MDS/AML. JCI Insight. 2021;6:e142149. 10.1172/jci.insight.142149 PMC7934867 33351783

[14] Cheng H , Zhang N , Pati D . Cohesin subunit RAD21: from biology to disease. Gene. 2020;758:144966. 32687945 10.1016/j.gene.2020.144966 PMC7949736

[15] Casa V , Moronta Gines M , Gade Gusmao E , Slotman JA , Zirkel A , Josipovic N , et al. Redundant and specific roles of cohesin STAG subunits in chromatin looping and transcriptional control. Genome Res. 2020;30:515–27. 32253279 10.1101/gr.253211.119 PMC7197483

[16] Chin CV , Antony J , Ketharnathan S , Labudina A , Gimenez G , Parsons KM , et al. Cohesin mutations are synthetic lethal with stimulation of WNT signaling. Elife. 2020;9:e61405. 33284104 10.7554/eLife.61405 PMC7746233

[17] Simonetti G , Mengucci C , Padella A , Fonzi E , Picone G , Delpino C , et al. Integrated genomic‐metabolic classification of acute myeloid leukemia defines a subgroup with NPM1 and cohesin/DNA damage mutations. Leukemia. 2021;35:2813–26. 34193978 10.1038/s41375-021-01318-x PMC8478658

[18] Jann JC , Tothova Z . Cohesin mutations in myeloid malignancies. Blood. 2021;138:649–61. 34157074 10.1182/blood.2019004259 PMC8394903

[19] van der Lelij P , Lieb S , Jude J , Wutz G , Santos CP , Falkenberg K , et al. Synthetic lethality between the cohesin subunits STAG1 and STAG2 in diverse cancer contexts. Elife. 2017;6:e26980. 28691904 10.7554/eLife.26980 PMC5531830

[20] Romero‐Pérez L , Surdez D , Brunet E , Delattre O , Grünewald TGP . STAG mutations in cancer. Trends Cancer. 2019;5:506–20. 31421907 10.1016/j.trecan.2019.07.001

[21] Coombes CE , Liu X , Abrams ZB , Coombes KR , Brock G . Simulation‐derived best practices for clustering clinical data. J Biomed Inform. 2021;118:103788. 33862229 10.1016/j.jbi.2021.103788 PMC9017600

[22] Luo J , Xie Y , Zheng Y , Wang C , Qi F , Hu J , et al. Comprehensive insights on pivotal prognostic signature involved in clear cell renal cell carcinoma microenvironment using the ESTIMATE algorithm. Cancer Med. 2020;9:4310–23. 32311223 10.1002/cam4.2983 PMC7300420

[23] Yoshihara K , Shahmoradgoli M , Martínez E , Vegesna R , Kim H , Torres‐Garcia W , et al. Inferring tumour purity and stromal and immune cell admixture from expression data. Nat Commun. 2013;4:2612. 24113773 10.1038/ncomms3612 PMC3826632

[24] Newman AM , Liu CL , Green MR , Gentles AJ , Feng W , Xu Y , et al. Robust enumeration of cell subsets from tissue expression profiles. Nat Methods. 2015;12:453–7. 25822800 10.1038/nmeth.3337 PMC4739640

[25] Liang ZQ , Gao L , Chen JH , Dai WB , Su YS , Chen G . Downregulation of the coiled‐coil domain containing 80 and its perspective mechanisms in ovarian carcinoma: a comprehensive study. Int J Genomics. 2021;2021:3752871. 34820451 10.1155/2021/3752871 PMC8608537

[26] Xu H , Ge Y , Liu Y , Zheng Y , Hu R , Ren C , et al. Identification of the key genes and immune infiltrating cells determined by sex differences in ischaemic stroke through co‐expression network module. IET Syst Biol. 2021;16:28–41. 34792838 10.1049/syb2.12039 PMC8849259

[27] Li Y , Deng Y , He J . Monocyte‐related gene biomarkers for latent and active tuberculosis. Bioengineered. 2021;12:10799–811. 34751089 10.1080/21655979.2021.2003931 PMC8809927

[28] Liang ZQ , Zhong LY , Li J , Shen JH , Tu XY , Zhong ZH , et al. Clinicopathological significance and underlying molecular mechanism of downregulation of basonuclin 1 expression in ovarian carcinoma. Exp Biol Med (Maywood). 2021;247:106–19. 34644201 10.1177/15353702211052036 PMC8777474

[29] Zeng D , Ye Z , Wu J , Zhou R , Fan X , Wang G , et al. Macrophage correlates with immunophenotype and predicts anti‐PD‐L1 response of urothelial cancer. Theranostics. 2020;10:7002–14. 32550918 10.7150/thno.46176 PMC7295060

[30] Jiang H , Ning G , Wang Y , Lv W . Identification of an m6A‐related signature as biomarker for hepatocellular carcinoma prognosis and correlates with sorafenib and anti‐PD‐1 immunotherapy treatment response. Dis Markers. 2021;2021:5576683. 34221187 10.1155/2021/5576683 PMC8213471

[31] Ye WY , Lu HP , Li JD , Chen G , He RQ , Wu HY , et al. Clinical implication of E2F transcription factor 1 in hepatocellular carcinoma tissues. Cancer Biother Radiopharm [Online ahead of print]. 2021. 10.1089/cbr.2020.4342 34619053

[32] Huang WY , Chen G , Chen SW , Dang YW , Deng Y , Zhou HF , et al. The indication of poor prognosis by high expression of ENO1 in squamous cell carcinoma of the lung. J Oncol. 2021;2021:9910962. 34504528 10.1155/2021/9910962 PMC8423576

[33] He RQ , Li JD , Du XF , Dang YW , Yang LJ , Huang ZG , et al. LPCAT1 overexpression promotes the progression of hepatocellular carcinoma. Cancer Cell Int. 2021;21:442. 34419067 10.1186/s12935-021-02130-4 PMC8380368

[34] Pang YY , Li JD , Gao L , Yang X , Dang YW , Lai ZF , et al. The clinical value and potential molecular mechanism of the downregulation of MAOA in hepatocellular carcinoma tissues. Cancer Med. 2020;9:8004–19. 32931665 10.1002/cam4.3434 PMC7643659

[35] Luna A , Elloumi F , Varma S , Wang Y , Rajapakse VN , Aladjem MI , et al. CellMiner cross‐database (CellMinerCDB) version 1.2: exploration of patient‐derived cancer cell line pharmacogenomics. Nucleic Acids Res. 2021;49:D1083–93. 33196823 10.1093/nar/gkaa968 PMC7779001

[36] Faubert B , Solmonson A , DeBerardinis RJ . Metabolic reprogramming and cancer progression. Science. 2020;368:eaaw5473. 32273439 10.1126/science.aaw5473 PMC7227780

[37] Zhang C , Yang M . The emerging factors and treatment options for NAFLD‐related hepatocellular carcinoma. Cancers (Basel). 2021;13:3740. 34359642 10.3390/cancers13153740 PMC8345138

[38] Nimitrungtawee N , Inmutto N , Chattipakorn SC , Chattipakorn N . Extracellular vesicles as a new hope for diagnosis and therapeutic intervention for hepatocellular carcinoma. Cancer Med. 2021;10:8253–71. 34708589 10.1002/cam4.4370 PMC8633266

[39] Yang W , Feng Y , Zhou J , Cheung OK , Cao J , Wang J , et al. A selective HDAC8 inhibitor potentiates antitumor immunity and efficacy of immune checkpoint blockade in hepatocellular carcinoma. Sci Transl Med. 2021;13:eaaz6804. 33827976 10.1126/scitranslmed.aaz6804

[40] Xu Q , Wang Y , Huang W . Identification of immune‐related lncRNA signature for predicting immune checkpoint blockade and prognosis in hepatocellular carcinoma. Int Immunopharmacol. 2021;92:107333. 33486322 10.1016/j.intimp.2020.107333

[41] Xu J , Zheng Q , Cheng X , Hu S , Zhang C , Zhou X , et al. Chemo‐photodynamic therapy with light‐triggered disassembly of theranostic nanoplatform in combination with checkpoint blockade for immunotherapy of hepatocellular carcinoma. J Nanobiotechnol. 2021;19:355. 10.1186/s12951-021-01101-1 PMC8557521 34717654

[42] Gu X , Guan J , Xu J , Zheng Q , Chen C , Yang Q , et al. Model based on five tumour immune microenvironment‐related genes for predicting hepatocellular carcinoma immunotherapy outcomes. J Transl Med. 2021;19:26. 33407546 10.1186/s12967-020-02691-4 PMC7788940

[43] Hung HC , Lee JC , Wang YC , Cheng CH , Wu TH , Lee CF , et al. Response prediction in immune checkpoint inhibitor immunotherapy for advanced hepatocellular carcinoma. Cancers (Basel). 2021;13:1607. 33807219 10.3390/cancers13071607 PMC8036568

[44] Pinato DJ , Marron TU , Mishra‐Kalyani PS , Gong Y , Wei G , Szafron D , et al. Treatment‐related toxicity and improved outcome from immunotherapy in hepatocellular cancer: evidence from an FDA pooled analysis of landmark clinical trials with validation from routine practice. Eur J Cancer. 2021;157:140–52. 34508996 10.1016/j.ejca.2021.08.020

[45] Pyatnitskiy MA , Arzumanian VA , Radko SP , Ptitsyn KG , Vakhrushev IV , Poverennaya EV , et al. Oxford nanopore MinION direct RNA‐seq for systems biology. Biology. 2021;10:1131. 34827124 10.3390/biology10111131 PMC8615092

[46] Liu T , Tan J , Wu M , Fan W , Wei J , Zhu B , et al. High‐affinity neoantigens correlate with better prognosis and trigger potent antihepatocellular carcinoma (HCC) activity by activating CD39(+)CD8(+) T cells. Gut. 2021;70:1965–77. 33262196 10.1136/gutjnl-2020-322196 PMC8458084

[47] Giannone G , Ghisoni E , Genta S , Scotto G , Tuninetti V , Turinetto M , et al. Immuno‐metabolism and microenvironment in cancer: key players for immunotherapy. Int J Mol Sci. 2020;21:4414. 10.3390/ijms21124414 PMC7352562 32575899

[48] Zhang B , Wu Q , Li B , Wang D , Wang L , Zhou YL . M(6)A regulator‐mediated methylation modification patterns and tumor microenvironment infiltration characterization in gastric cancer. Mol Cancer. 2020;19:53. 32164750 10.1186/s12943-020-01170-0 PMC7066851

[49] Carico ZM , Stefan HC , Justice M , Yimit A , Dowen JM . A cohesin cancer mutation reveals a role for the hinge domain in genome organization and gene expression. PLoS Genet. 2021;17:e1009435. 33760811 10.1371/journal.pgen.1009435 PMC7990204

[50] Antony J , Chin CV , Horsfield JA . Cohesin mutations in cancer: emerging therapeutic targets. Int J Mol Sci. 2021;22:6788. 34202641 10.3390/ijms22136788 PMC8269296

[51] Martín‐Izquierdo M , Abáigar M , Hernández‐Sánchez JM , Tamborero D , López‐Cadenas F , Ramos F , et al. Co‐occurrence of cohesin complex and Ras signaling mutations during progression from myelodysplastic syndromes to secondary acute myeloid leukemia. Haematologica. 2021;106:2215–23. 32675227 10.3324/haematol.2020.248807 PMC8327724

[52] Yang C , Huang X , Li Y , Chen J , Lv Y , Dai S . Prognosis and personalized treatment prediction in TP53‐mutant hepatocellular carcinoma: an in silico strategy towards precision oncology. Brief Bioinform. 2021;22:bbaa164. 32789496 10.1093/bib/bbaa164

[53] Patel SA , Lloyd MR , Cerny J , Shi Q , Simin K , Ediriwickrema A , et al. Clinico‐genomic profiling and clonal dynamic modeling of TP53‐aberrant myelodysplastic syndrome and acute myeloid leukemia. Leuk Lymphoma. 2021;62:3348–60. 34496723 10.1080/10428194.2021.1957869

[54] Anazawa Y , Arakawa H , Nakagawa H , Nakamura Y . Identification of STAG1 as a key mediator of a p53‐dependent apoptotic pathway. Oncogene. 2004;23:7621–7. 15361841 10.1038/sj.onc.1207270

[55] Pan H , Jin M , Ghadiyaram A , Kaur P , Miller HE , Ta HM , et al. Cohesin SA1 and SA2 are RNA binding proteins that localize to RNA containing regions on DNA. Nucleic Acids Res. 2020;48:5639–55. 32352519 10.1093/nar/gkaa284 PMC7261166

[56] Vodicka P , Andera L , Opattova A , Vodickova L . The interactions of DNA repair, telomere homeostasis, and p53 mutational status in solid cancers: risk, prognosis, and prediction. Cancer. 2021;13:479. 10.3390/cancers13030479 PMC7865496 33513745

[57] Song S , Shi Y , Wu W , Wu H , Chang L , Peng P , et al. Reticulon 3‐mediated Chk2/p53 activation suppresses hepatocellular carcinogenesis and is blocked by hepatitis B virus. Gut. 2021;70:2159–71. 33303565 10.1136/gutjnl-2020-321386

[58] Mengying C . The function and regulatory mechanism of microRNA‐23a‐27a‐24‐2 cluster in hepatocellular carcinoma, Jilin University. (In Chinese)

[59] Schmidt D , Schwalie PC , Ross‐Innes CS , Hurtado A , Brown GD , Carroll JS , et al. A CTCF‐independent role for cohesin in tissue‐specific transcription. Genome Res. 2010;20:578–88. 20219941 10.1101/gr.100479.109 PMC2860160

[60] Kasowski M , Kyriazopoulou‐Panagiotopoulou S , Grubert F , Zaugg JB , Kundaje A , Liu Y , et al. Extensive variation in chromatin states across humans. Science (New York, NY). 2013;342:750–2. 10.1126/science.1242510 PMC4075767 24136358

[61] Steiner LA , Schulz V , Makismova Y , Lezon‐Geyda K , Gallagher PG . CTCF and CohesinSA‐1 mark active promoters and boundaries of repressive chromatin domains in primary human erythroid cells. PLoS ONE. 2016;11:e0155378. 27219007 10.1371/journal.pone.0155378 PMC4878738

[62] Zhang N , Coutinho LE , Pati D . PDS5A and PDS5B in Cohesin function and human disease. Int J Mol Sci. 2021;22:5868. 34070827 10.3390/ijms22115868 PMC8198109

[63] Morales C , Ruiz‐Torres M , Rodríguez‐Acebes S , Lafarga V , Rodríguez‐Corsino M , Megías D , et al. PDS5 proteins are required for proper cohesin dynamics and participate in replication fork protection. J Biol Chem. 2020;295:146–57. 31757807 10.1074/jbc.RA119.011099 PMC6952610

[64] Al‐Jomah N , Mukololo L , Anjum A , Al Madadha M , Patel R . Pds5A and Pds5B display non‐redundant functions in mitosis and their loss triggers Chk1 activation. Front Cell Dev Biol. 2020;8:531. 32760717 10.3389/fcell.2020.00531 PMC7372117

[65] Ketharnathan S , Labudina A , Horsfield JA . Cohesin components Stag1 and Stag2 differentially influence Haematopoietic mesoderm development in zebrafish embryos. Front Cell Dev Biol. 2020;8:617545. 33365313 10.3389/fcell.2020.617545 PMC7750468

[66] Zhang S , Liu W , Yang Y , Sun K , Li S , Xu H , et al. TMEM30A deficiency in endothelial cells impairs cell proliferation and angiogenesis. J Cell Sci. 2019;132:jcs225052. 30814335 10.1242/jcs.225052

[67] Bartoletti G , Dong C , Umar M , He F . Pdgfra regulates multipotent cell differentiation towards chondrocytes via inhibiting Wnt9a/beta‐catenin pathway during chondrocranial cartilage development. Dev Biol. 2020;466:36–46. 32800757 10.1016/j.ydbio.2020.08.004 PMC7494641

[68] Hayashi T , Yamamoto S , Hamashima T , Mori H , Sasahara M , Kuroda S . Critical role of platelet‐derived growth factor‐α in angiogenesis after indirect bypass in a murine moyamoya disease model. J Neurosurg. 2020;134:1535–43. 32442967 10.3171/2020.3.JNS193273

[69] Lin LH , Lin JS , Yang CC , Cheng HW , Chang KW , Liu CJ . Overexpression of platelet‐derived growth factor and its receptor are correlated with Oral tumorigenesis and poor prognosis in Oral squamous cell carcinoma. Int J Mol Sci. 2020;21:2360. 10.3390/ijms21072360 PMC7177415 32235327

[70] Zhu K , Pan Q , Zhang X , Kong LQ , Fan J , Dai Z , et al. MiR‐146a enhances angiogenic activity of endothelial cells in hepatocellular carcinoma by promoting PDGFRA expression. Carcinogenesis. 2013;34:2071–9. 23671131 10.1093/carcin/bgt160

[71] Shao QP , Wei C , Yang J , Zhang WZ . miR‐3609 decelerates the clearance of sorafenib in hepatocellular carcinoma cells by targeting EPAS‐1 and reducing the activation of the Pregnane X receptor pathway. Onco Targets Ther. 2020;13:7213–27. 32801751 10.2147/OTT.S246471 PMC7394586

[72] Kato H , Ito E , Shi W , Alajez NM , Yue S , Lee C , et al. Efficacy of combining GMX1777 with radiation therapy for human head and neck carcinoma. Clin Cancer Res. 2010;16:898–911. 20103674 10.1158/1078-0432.CCR-09-1945

