# Supplementary material for: A cohesin‐associated gene score may predict immune checkpoint blockade in hepatocellular carcinoma
Source: FEBS Open Bio. 2022 Sep 2;12(10):1857–74. doi: 10.1002/2211-5463.13474 (PMC9527596; doi:10.1002/2211-5463.13474)
Supplement: Supplementary file 1 — Fig. S1. Design route of this research. Fig. S2. Metabolic characterization and mutation co‐occurrence statuses of HCC patients. Based on the identified CAG clusters, the metabolic characterization and mutation co‐occurrence statuses of different HCC patients were compared. (A–C) The HCC patients in CAG cluster 2 with poor prognosis displayed prominently lower metabolism levels of drug, retinol, and xenobiotics than the HCC patients in the other CAG clusters. Panels D–F show the mutation co‐occurrence statuses of different CAG patterns. TP53 mutation co‐occurrence was more frequent in the HCC patients in the CAG cluster 2 than in the patients in the other CAG clusters. Abbreviations: HCC, hepatocellular carcinoma; CAG, cohesin‐associated gene. Fig. S3. Prognostic prediction ability of STAG1 in patient receiving immunotherapy. We appraised the prognostic value of STAG1 in the IMvigor210 urothelial cancer (A) and GSE78220 melanoma (B) immunotherapy cohorts. Higher STAG1 expression level predicted better overall survival outcomes in patients who received immunotherapy. Fig. S4. Upregulation of STAG1 based on global HCC data. A total of 37 platform matrices were enrolled to analyze the overall expression status of STAG1 in HCC. (A) The standard mean difference forest plot indicated that STAG1 was upregulated in 3313 HCC tissue samples in comparison to 2692 non‐HCC tissue samples. (B) The sensitivity analysis plot indicated that the included datasets could not explain the major source of heterogeneity. (C) The funnel plot implied insignificant publication bias, which showed the stability of the quantitative synthesis result (Begg's test: P value = 0.067). (D) The summary characteristics operating curve showed a moderate discriminatory ability of STAG1, with weak sensitivity (E) and moderate specificity (F). (G–I) Fagan's nomogram and likelihood ratio forest plots indicated the general accuracy of STAG1 in differentiating between HCC and non‐HCC tissue samples. Abbreviati [file FEB4-12-1857-s001.docx]

**Supplementary Table 1: Overexpressed STAG1 in 37 hepatocellular carcinoma platform matrices**

| **ID** | **N _HCC_** | **M _HCC_** | **SD _HCC_** | **N _control_** | **M _control_** | **SD _control_** |
| --- | --- | --- | --- | --- | --- | --- |
| E_MTAB_8887 | 23 | 3.05 | 0.63 | 17 | 3.26 | 0.89 |
| GPL11154 | 163 | 2.61 | 0.92 | 140 | 2.78 | 0.64 |
| GPL14951 | 93 | 9.78 | 1.13 | 18 | 9.62 | 1.53 |
| GPL16043 | 25 | 1.23 | 0.59 | 25 | 1.23 | 0.47 |
| GPL16791 | 79 | 5.78 | 1.02 | 78 | 5.63 | 0.86 |
| GPL21047 | 10 | 2.90 | 0.09 | 10 | 2.86 | 0.06 |
| GPL5175 | 48 | 3.08 | 0.07 | 48 | 3.03 | 0.05 |
| GPL570 | 844 | 4.17 | 0.17 | 528 | 4.05 | 0.13 |
| GPL571 | 96 | 2.86 | 0.09 | 131 | 2.84 | 0.06 |
| GPL6244 | 66 | 3.60 | 0.13 | 75 | 3.56 | 0.11 |
| GPL6480 | 83 | 2.75 | 0.13 | 82 | 2.69 | 0.13 |
| GPL6947 | 104 | 2.71 | 0.25 | 97 | 2.87 | 0.22 |
| GPL9052 | 60 | 3.18 | 0.45 | 60 | 2.93 | 0.34 |
| GSE115018_GPL20115 | 12 | -1.17 | 0.55 | 12 | -1.40 | 0.30 |
| GSE124535_GPL20795 | 35 | 2.82 | 0.56 | 35 | 1.96 | 0.33 |
| GSE125469_GPL20301 | 3 | 5.75 | 0.07 | 3 | 4.77 | 0.09 |
| GSE128274_GPL18573 | 4 | 6.70 | 0.29 | 4 | 6.25 | 0.15 |
| GSE14520_GPL3921 | 225 | 2.77 | 0.11 | 220 | 2.63 | 0.06 |
| GSE166163_GPL23126 | 3 | 5.80 | 0.35 | 3 | 6.03 | 1.08 |
| GSE20140_GPL18461 | 35 | 7.80 | 0.25 | 34 | 7.65 | 0.18 |
| GSE22058_GPL6793 | 100 | 7.94 | 0.37 | 97 | 7.91 | 0.22 |
| GSE22405_GPL10553 | 24 | 2.86 | 0.11 | 24 | 2.78 | 0.07 |
| GSE25097_GPL10687 | 268 | 2.32 | 0.01 | 289 | 2.32 | 0.01 |
| GSE33294_GPL10999 | 3 | 3.29 | 0.23 | 3 | 1.98 | 0.10 |
| GSE46408_GPL4133 | 6 | 8.36 | 0.44 | 6 | 7.20 | 0.47 |
| GSE46444_GPL13369 | 88 | 8.82 | 1.61 | 48 | 8.90 | 1.85 |
| GSE50579_GPL14550 | 67 | 2.89 | 0.09 | 10 | 2.69 | 0.14 |
| GSE54238_GPL16955 | 26 | 7.71 | 1.30 | 30 | 7.95 | 0.77 |
| GSE55048_GPL9115 | 4 | 2.31 | 0.32 | 4 | 1.46 | 0.38 |
| GSE56545_GPL15433 | 21 | 3.33 | 0.07 | 21 | 3.29 | 0.03 |
| GSE57555_GPL16699 | 5 | -0.18 | 0.02 | 5 | -0.15 | 0.03 |
| GSE59259_GPL18451 | 8 | 9.19 | 0.52 | 8 | 9.48 | 0.27 |
| GSE60502_GPL96 | 18 | 8.76 | 0.24 | 18 | 8.24 | 0.19 |
| GSE63898_GPL13667 | 228 | 6.47 | 0.40 | 168 | 6.32 | 0.23 |
| GSE67764_GPL17077 | 3 | -1.76 | 0.15 | 6 | -1.82 | 0.07 |
| GSE76311_GPL17586 | 62 | 2.93 | 0.07 | 59 | 2.85 | 0.03 |
| TCGA_GTEx_liver | 371 | 2.37 | 0.69 | 276 | 2.18 | 0.38 |
| **In total** | **3313** |  |  | **2692** |  |  |

Footnote: N, number; SD, standard deviation; M, mean.

**Supplementary Table 2: Fundamental dataset information of the included global hepatocellular carcinoma datasets**

| **Platform ID** | **Dataset** |
| --- | --- |
| Agilent SurePrint G3 Human GE v2 8x60k | E_MTAB_8887 Germany |
| GPL11154 | GSE114564 South Korea, GSE148355 South Korea, GSE63863 China, GSE65485 China, GSE73708 USA, GSE81550 USA, GSE87592 China |
| GPL14951 | GSE57727 Spain, GSE98617 Spain |
| GPL16043 | GSE113996 China, GSE74656 China |
| GPL16791 | GSE104310 China, GSE63018 USA, GSE77509 China, GSE94660 USA, GSE97214 China, GSE140845 China, GSE112221 USA |
| GPL21047 | GSE101728 China, GSE98269-GPL21047 China |
| GPL5175 | GSE12941 Japan, GSE84005 China |
| GPL570 | GSE101685 China, GSE102079 Japan, GSE107170 Italy, GSE112790 Japan, GSE121248 Singapore, GSE17548 Turkey, GSE19665 Japan, GSE29721 Canada, GSE33006 China, GSE41804 Japan, GSE45436 China, GSE6222 China, GSE62232 France, GSE6764 USA, GSE99807 China, GSE84402 China |
| GPL571 | GSE14323-GPL571 USA, GSE14520-GPL571 USA, GSE17967 USA, GSE9839 Switzerland |
| GPL6244 | GSE45050 USA, GSE64041 Switzerland |
| GPL6480 | GSE117361 China, GSE54236 Italy |
| GPL6947 | GSE87630 South Korea, GSE89377 South Korea |
| GPL9052 | GSE25599 China, GSE77314 China |
| GPL20115 | GSE115018 China |
| GPL20795 | GSE124535 China |
| GPL20301 | GSE125469 China |
| GPL18573 | GSE128274 China |
| GPL3921 | GSE14520-GPL3921 USA |
| GPL23126 | GSE166163 China |
| GPL18461 | GSE20140 USA |
| GPL6793 | GSE22058 USA |
| GPL10553 | GSE22405 USA |
| GPL10687 | GSE25097 USA |
| GPL10999 | GSE33294 China |
| GPL4133 | GSE46408 China |
| GPL13369 | GSE46444 USA |
| GPL14550 | GSE50579 Germany |
| GPL16955 | GSE54238 USA |
| GPL9115 | GSE55048 China |
| GPL15433 | GSE56545 USA |
| GPL16699 | GSE57555 Japan |
| GPL18451 | GSE59259 Italy |
| GPL96 | GSE60502 China |
| GPL13667 | GSE63898 USA |
| GPL17077 | GSE67764 China |
| GPL17586 | GSE76311 USA |
| TCGA-GTEx liver | / |


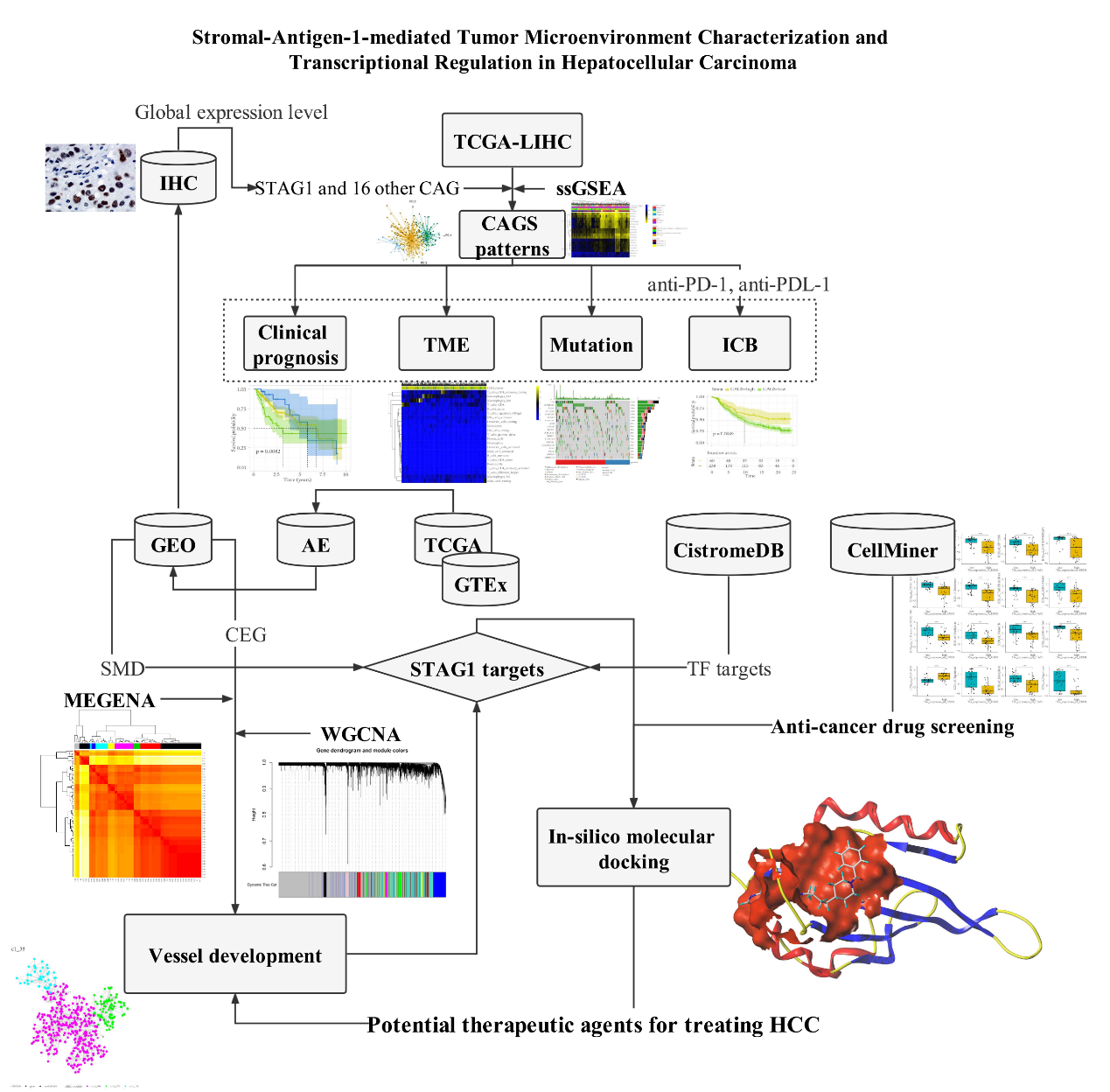


**Supplementary Figure 1: Design route of this research**





**Supplementary Figure 2: Metabolic characterization and mutation co-occurrence statuses of HCC patients**

Based on the identified CAG clusters, the metabolic characterization and mutation co-occurrence statuses of different HCC patients were compared. (A–C) The HCC patients in CAG cluster 2 with poor prognosis displayed prominently lower metabolism levels of drug, retinol, and xenobiotics than the HCC patients in the other CAG clusters. Panels D–F show the mutation co-occurrence statuses of different CAG patterns. *TP53* mutation co-occurrence was more frequent in the HCC patients in the CAG cluster 2 than in the patients in the other CAG clusters. Abbreviations: HCC, hepatocellular carcinoma; CAG, cohesin-associated gene.


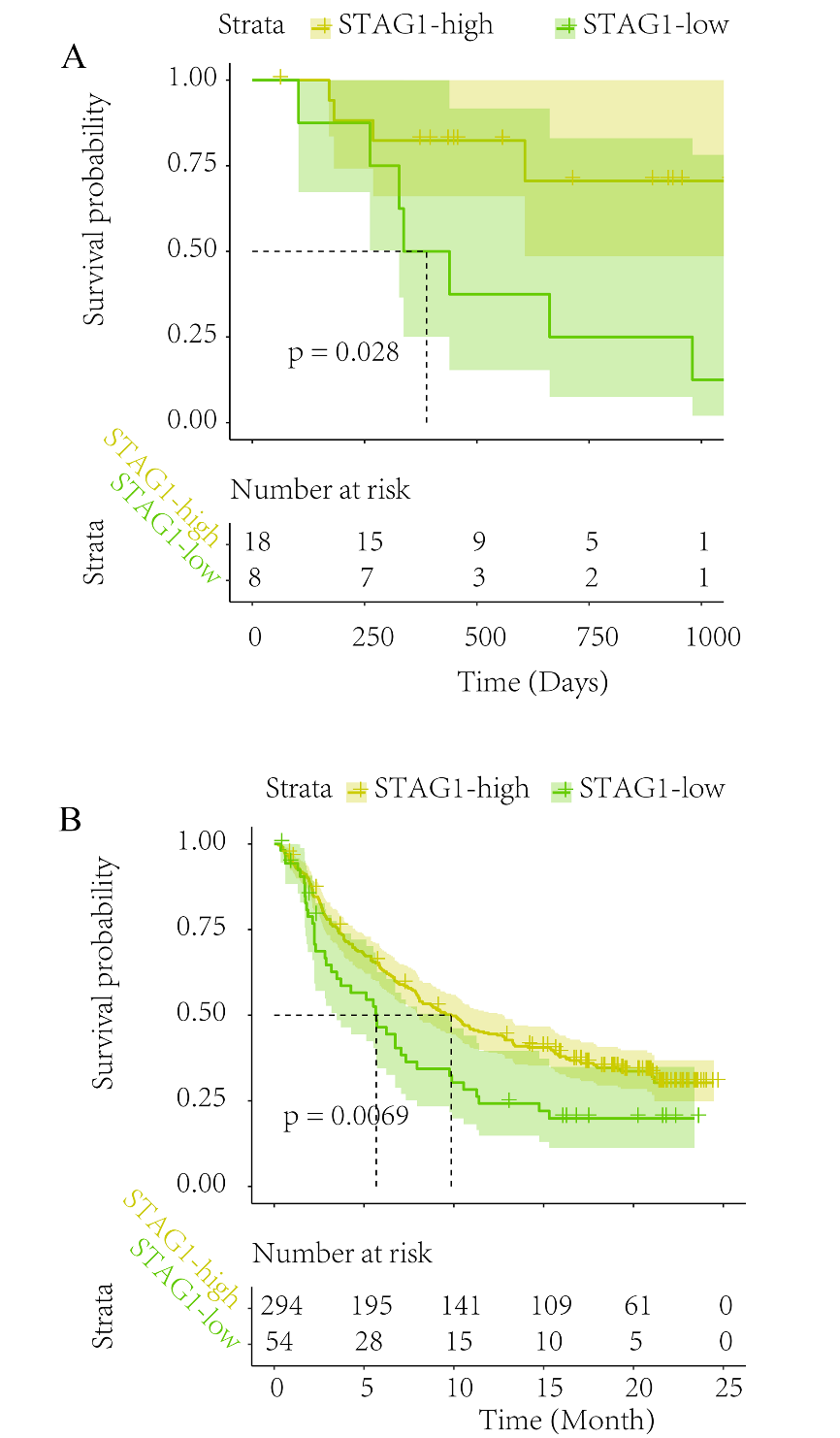


**Supplementary Figure 3: Prognostic prediction ability of STAG1 in patient receiving immunotherapy**

We appraised the prognostic value of STAG1 in the IMvigor210 urothelial cancer (A) and GSE78220 melanoma (B) immunotherapy cohorts. Higher STAG1 expression level predicted better overall survival outcomes in patients who received immunotherapy.





**Supplementary Figure 4: Upregulation of STAG1 based on global HCC data**

A total of 37 platform matrices were enrolled to analyze the overall expression status of STAG1 in HCC. (A) The standard mean difference forest plot indicated that STAG1 was upregulated in 3313 HCC tissues in comparison to 2692 non-HCC tissues. (B) The sensitivity analysis plot indicated that the included datasets could not explain the major source of heterogeneity. (C) The funnel plot implied insignificant publication bias, which showed the stability of the quantitative synthesis result (Begg’s test: *P* value = .067). (D) The summary characteristics operating curve showed a moderate discriminatory ability of STAG1, with weak sensitivity (E) and moderate specificity (F). (G–I) Fagan’s nomogram and likelihood ratio forest plots indicated the general accuracy of STAG1 in differentiating between HCC and non-HCC tissues. Abbreviation: HCC, hepatocellular carcinoma.


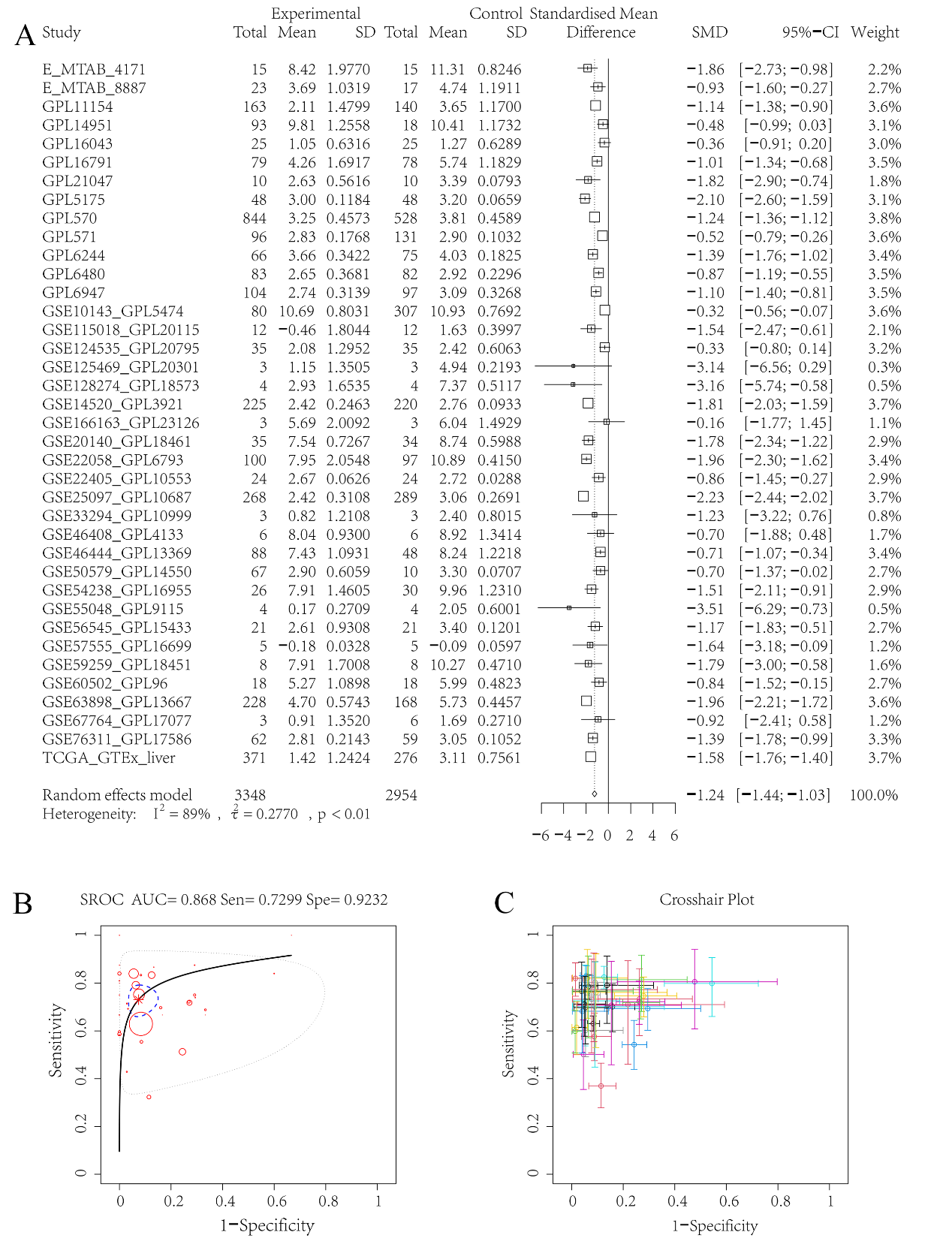


**Supplementary Figure 5: Comprehensive mRNA expression level of PDGFRA in the HCC tissue samples**

PDGFRA was significantly downregulated in the HCC tissue samples when compared with normal liver tissue specimens (A). Downregulated PDGFRA mRNA showed a moderate discriminatory ability between HCC and normal liver tissue samples (B, C). Abbreviation: HCC, hepatocellular carcinoma.


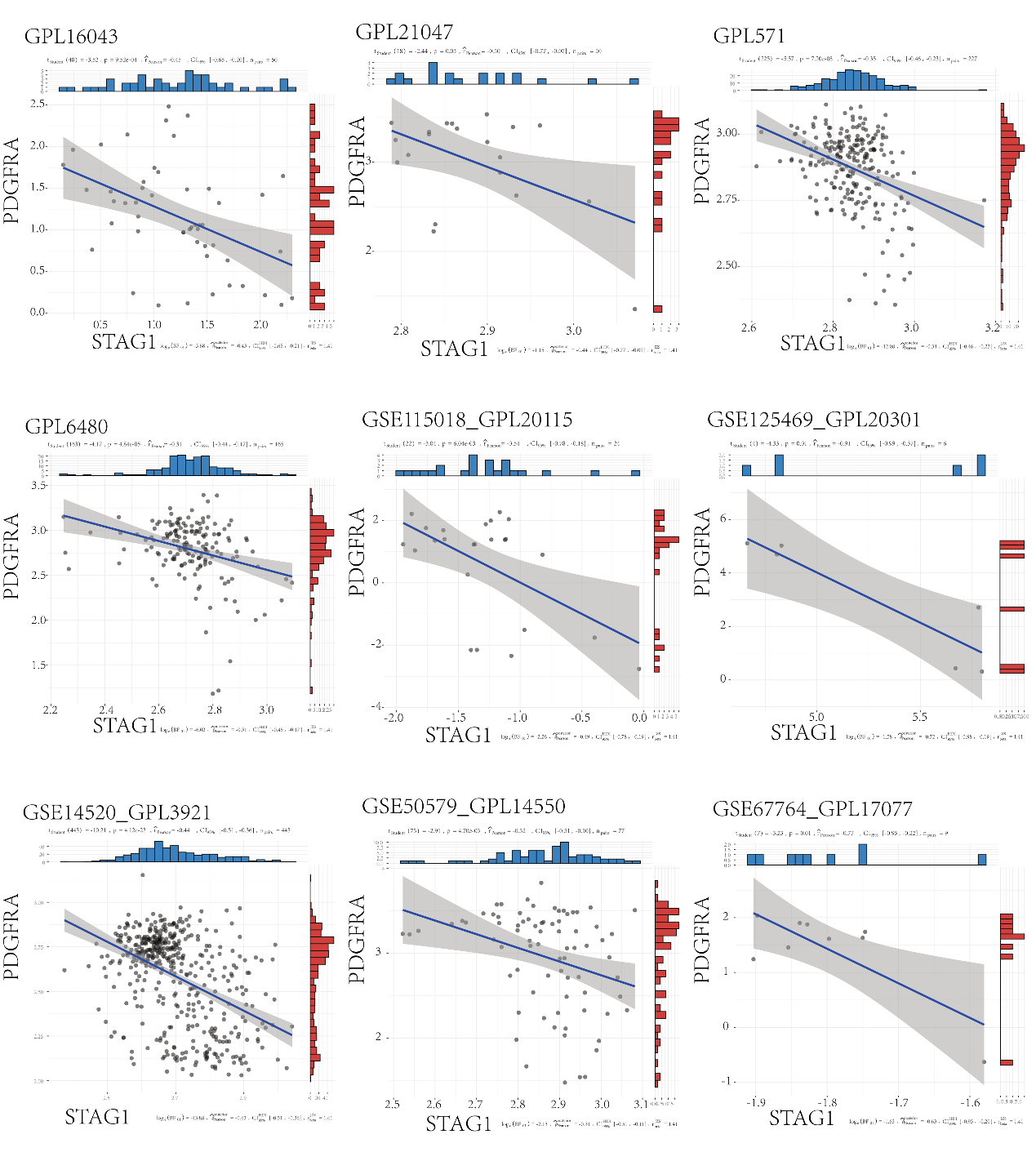


**Supplementary Figure 6: Negative correlations between STAG1 factor and PDGFRA target**

We computed the Pearson correlation coefficients between STAG1 expression level and PDGFRA expression level. STAG1 expression level was inversely correlated to PDGFRA mRNA level.


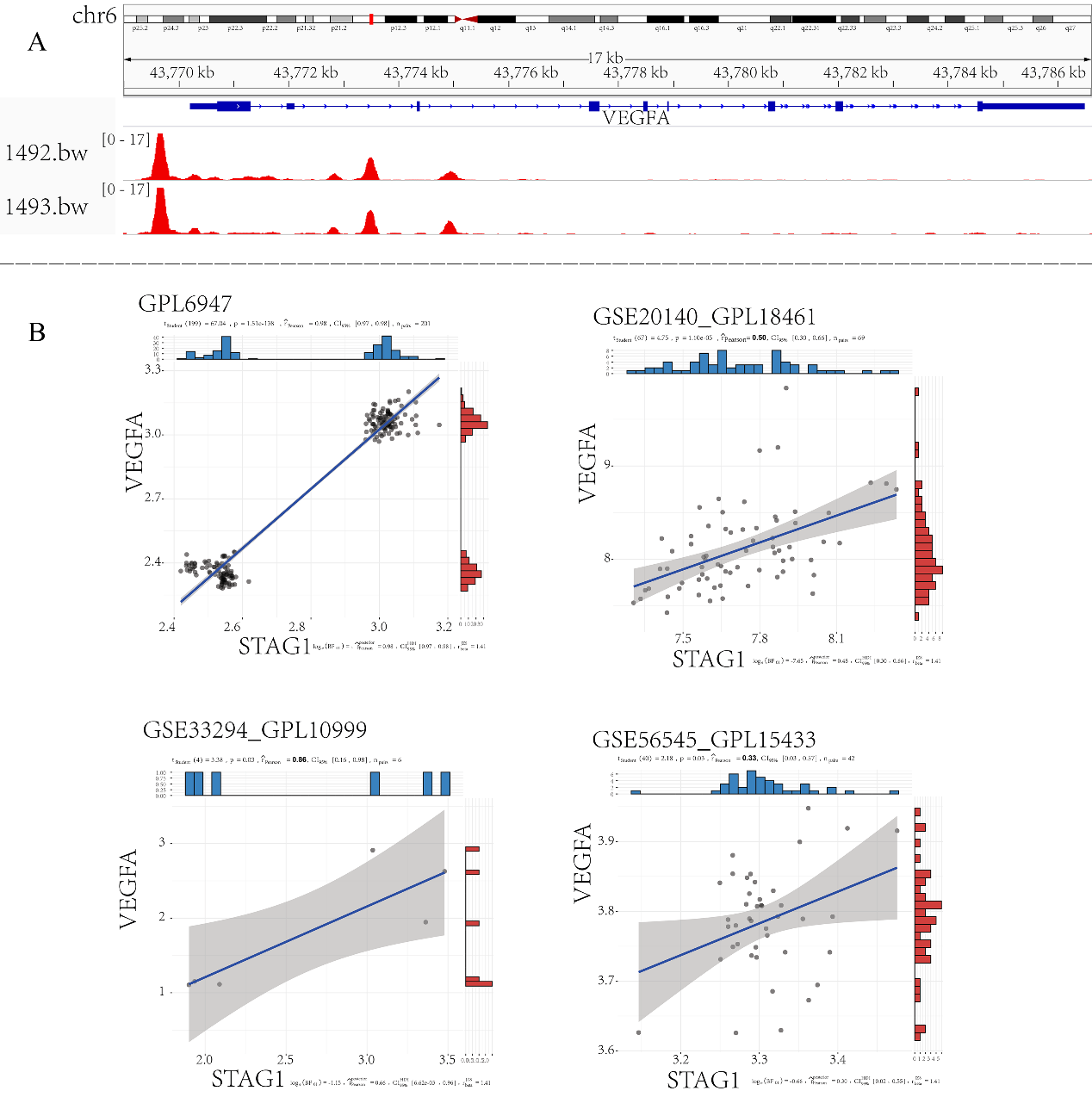


**Supplementary Figure 7: Potential correlations between STAG1 factor and** **the VEGFA mRNA expression**

Chromatin immunoprecipitation sequencing data were reanalyzed to explore the potential regulation between STAG1 and vascular endothelial growth factor A (VEGFA). STAG1 showed an obvious transcriptional factor binding intensity in the promoter region of VEGFA (A). STAG1 was positively correlated to the expression level of VEGFA (B).


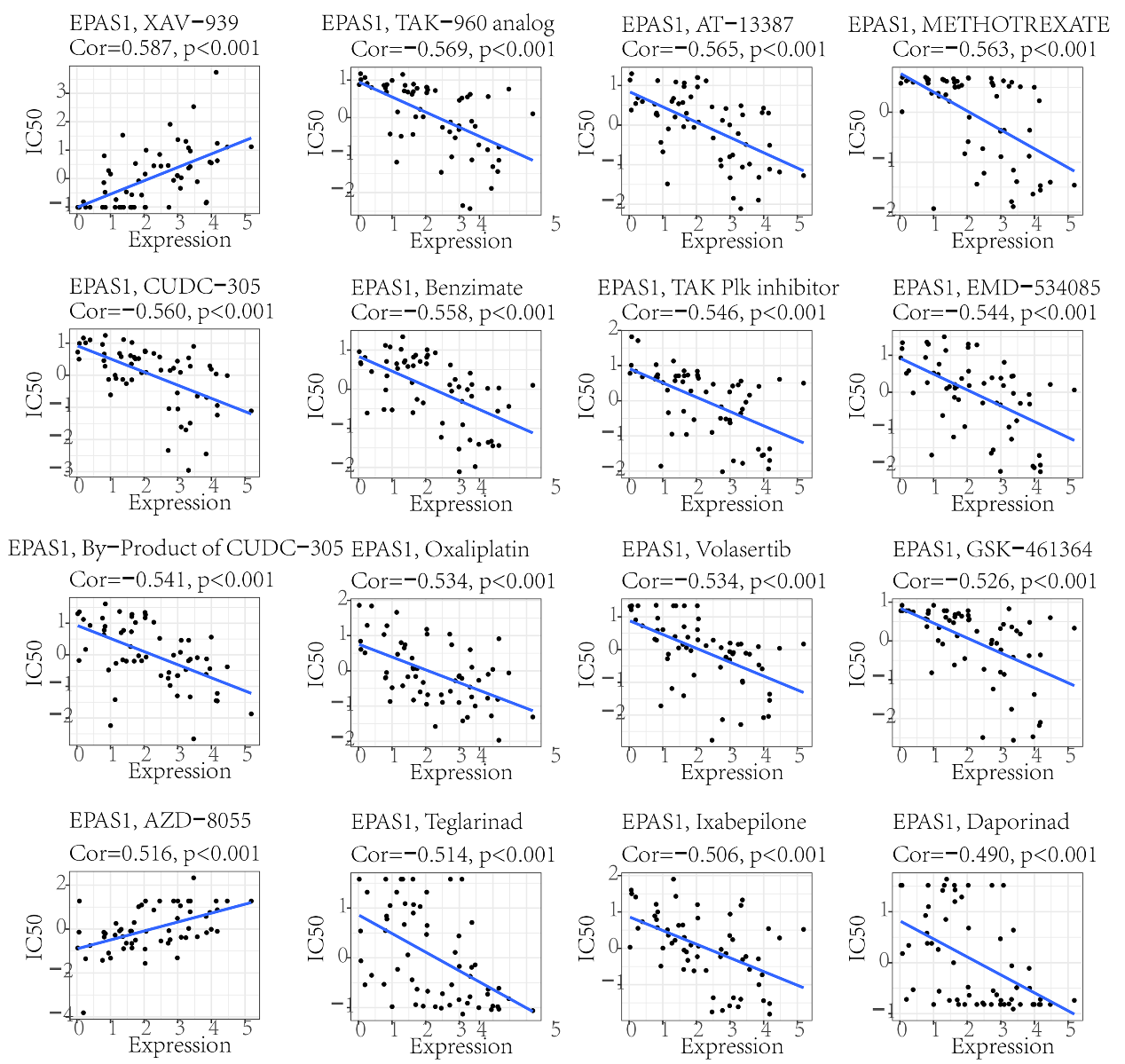


**Supplementary Figure 8: Potential correlations between EPAS1 expression and** **the sensitivity of anti-cancer agents**

We computed Pearson correlation coefficients between EPAS1 expression and half maximal inhibitory concentration of the screened agents.


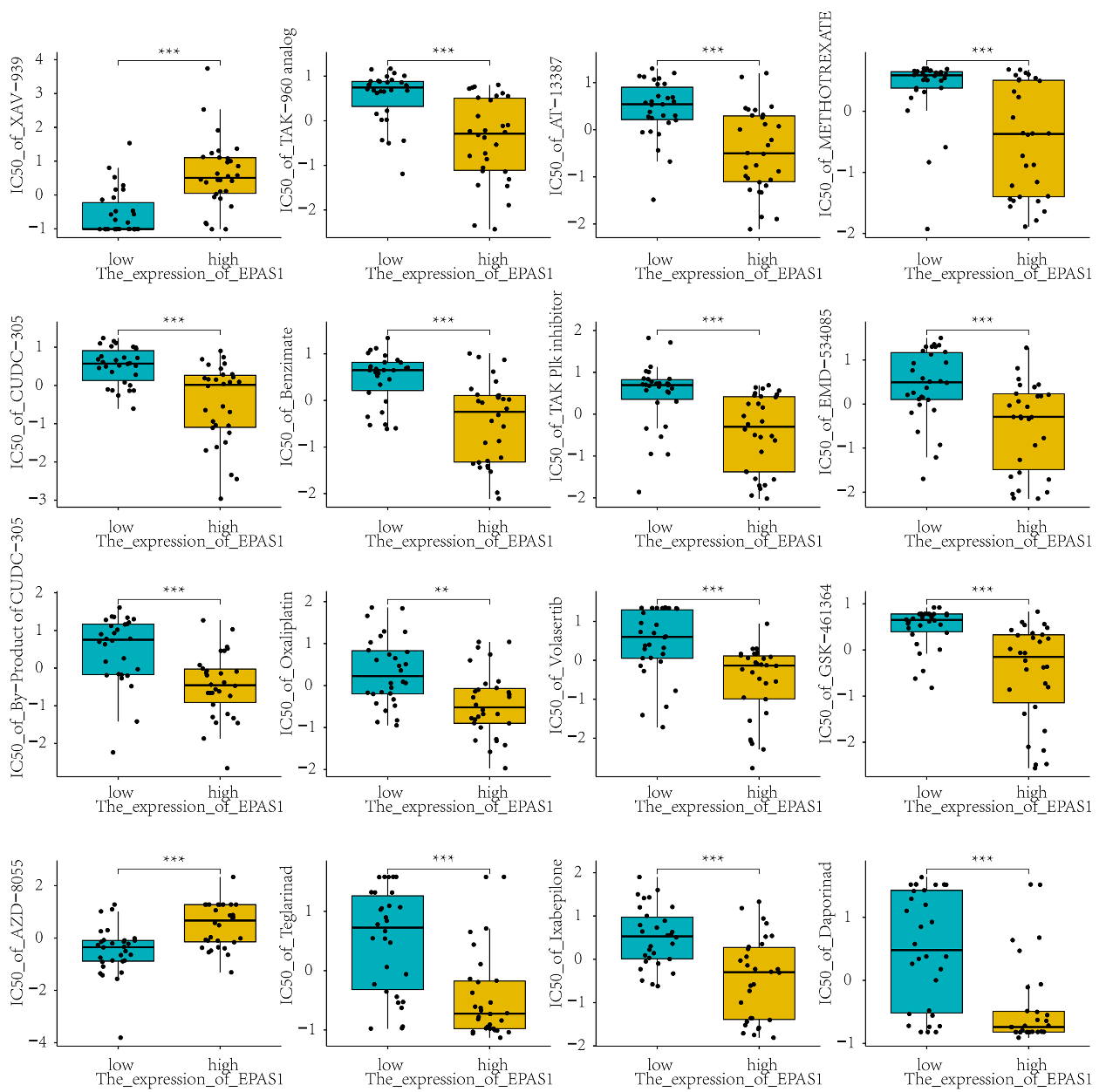


**Supplementary Figure 9: The discrepancies of half maximal inhibitory concentration in the EPAS1-high expression group and the EPAS1-low expression group.**

Note: A Wilcoxon test was conducted to compare the discrepancies of half maximal inhibitory concentration between EPAS1-high expression group (n=30) and the EPAS1-low expression group (n=30). ***, *P* value<0.001.
